# Activities of daily living and their neural correlates across the Alzheimer's disease continuum: Evidence from a Latin American cohort

**DOI:** 10.1002/alz.71445

**Published:** 2026-04-29

**Authors:** Fernando Henríquez, Patricio Riquelme, Gonzalo Forno, Joaquín Migeot, Rodrigo Henríquez, Patricia Lillo, Daniela Thumala‐Dockendorff, Cecilia Okuma, Cecilia González Campo, Michael Hornberger, Francisco Aboitiz, Andrea Slachevsky

**Affiliations:** ^1^ Neuropsychology and Clinical Neuroscience Laboratory (LANNEC) Physiopathology Department Institute of Biomedical Sciences (ICBM) Faculty of Medicine University of Chile Santiago Chile; ^2^ Geroscience Center for Brain Health and Metabolism (GERO) Santiago Chile; ^3^ Interdisciplinary Center for Neuroscience (NeuroUC) Laboratory for Cognitive and Evolutionary Neuroscience (LANCE) Department of Psychiatry Faculty of Medicine Pontificia Universidad Católica de Chile Santiago Chile; ^4^ Memory and Neuropsychiatric Center (CMYN) Neurology Department Hospital del Salvador & Faculty of Medicine University of Chile Santiago Chile; ^5^ Medical Technology Department Faculty of Medicine University of Chile Santiago Chile; ^6^ Latin American Brain Health Institute (BrainLat) Universidad Adolfo Ibáñez Santiago Chile; ^7^ Trinity College Dublin Global Brain Health Institute (GBHI) Dublin UK; ^8^ Interdisciplinary Center for Neuroscience (NeuroUC) Laboratory for Cognitive and Evolutionary Neuroscience (LANCE) Department of Neurology Faculty of Medicine Pontificia Universidad Católica de Chile Santiago Chile; ^9^ Centro ANID de Interés Nacional para Investigación e Innovación en Niñez, Adolescencia, Resiliencia y Adversidad IINARA Chile; ^10^ Neurology Department South Division Faculty of Medicine University of Chile Santiago Chile; ^11^ Neurology Unit Hospital San José Santiago Chile; ^12^ Institute of Neurosurgery Dr. Alfonso Asenjo Santiago Chile; ^13^ Cognitive Neuroscience Center (CNC) Universidad de San Andrés Buenos Aires Argentina; ^14^ National Scientific and Technical Research Council (CONICET) Buenos Aires Argentina; ^15^ Clinical Neurosciences, Clinical and Experimental Sciences Faculty of Medicine University of Southampton Southampton UK; ^16^ Neurology and Psychiatry Department Clínica Alemana–Universidad del Desarrollo Santiago Chile

**Keywords:** activities of daily living, Alzheimer's disease continuum, brain atrophy, functional ability, neural correlates, voxel‐based morphometry

## Abstract

**INTRODUCTION:**

Functional decline in activities of daily living (ADL)—advanced (AADL), instrumental (IADL), and basic (BADL)—is a hallmark of Alzheimer's disease (AD). However, integrated clinical–neuroanatomical evidence on progression across the AD continuum remains limited, particularly in Latin American populations.

**METHODS:**

We studied 138 older adults with subjective cognitive complaints (SCCs), mild cognitive impairment (MCI), and Alzheimer's disease dementia (ADD). ADL domains were assessed using the Technology‐Activities of Daily Living Questionnaire. Structural magnetic resonance imaging data were analyzed using voxel‐based morphometry (VBM) to identify gray matter (GM) correlates of ADL performance.

**RESULTS:**

A hierarchical decline from AADL to IADL to BADL differentiated clinical stages. SCC and MCI differed mainly in AADL performance, whereas ADD showed decline across all domains. VBM revealed GM correlates consistent with this hierarchy, with distinct but partially overlapping substrates for each ADL domain.

**DISCUSSION:**

These findings underscore a diagnostically informative and anatomically organized progression of ADL decline.

## Highlights

- Advanced activities of daily living (AADL) showed early variability and decline, supporting their utility as sensitive markers of initial functional deterioration across the Alzheimer's disease (AD) continuum.
- Gray matter atrophy patterns, both exclusive and overlapping, were associated with AADL, instrumental activities of daily living, and basic activities of daily living, revealing stage‐specific neuroanatomical substrates.
- A multidimensional framework integrating the hierarchical complexity of activities of daily living with structural neuroimaging enhances the characterization of functional phenotypes across the AD continuum.
- Findings from a Latin American cohort extend evidence to underrepresented populations and highlight the need for context‐sensitive functional assessments.

## 1 BACKGROUND

Alzheimer's disease (AD), the most common cause of dementia, is a neurodegenerative disorder characterized by progressive cognitive and functional decline, leading to loss of independence in everyday life.^1^ The AD pathophysiological process begins decades before clinical symptoms appear, supporting its conceptualization as a continuum spanning from an at‐risk stage, often marked by subjective cognitive complaints (SCCs), through prodromal mild cognitive impairment (MCI), and finally to AD dementia (ADD).^2^

Functional ability, reflected in the capacity to perform activities of daily living (ADL), is a critical clinical outcome in AD.^3^ Decline in ADLs is associated with worse prognosis, faster disease progression,^4^ and increased emotional and financial burden on families.^5^ ADLs are commonly organized into a hierarchical set of domains of increasing complexity: basic ADLs (BADL), related to self‐care; instrumental ADLs (IADL), necessary for independent functioning; and advanced ADLs (AADL), involving complex social, cognitive, and role‐based activities.^6^

Functional decline, traditionally considered a late feature of the AD continuum,^7^ is now recognized as emerging years before dementia onset.^8^ Individuals with MCI frequently show reduced accuracy and efficiency in performing complex ADLs, particularly IADLs and AADLs.^9^, ^10^ Early functional decline has also been observed in cognitively unimpaired (CU) individuals, especially those with AD‐related biomarkers or SCC, and is associated with an increased risk of progression to ADD.^11^ Most existing studies have focused primarily on IADLs and typically compared pairs of clinical stages (CU vs. SCC, CU vs. MCI, or MCI vs. ADD).^11^, ^12^, ^13^ Fewer studies have examined multiple stages simultaneously (CU, MCI, ADD), and these have often evaluated only IADLs and BADLs^14^ or have exclusively targeted AADLs.^9^ Thus, comprehensive assessments encompassing all ADL domains across the disease continuum remain limited, despite their value for early detection, monitoring, and evaluation of disease impact.

A parallel gap exists in neuroanatomical research. Most studies examining associations between IADL decline and brain atrophy have compared CU and MCI or ADD.^15^, ^16^ Some have included both BADLs and IADLs, contrasting CU with ADD^17^ or focusing solely on ADD.^18^ Few investigations have assessed multiple disease stages (SCC/CU, MCI, ADD), typically limited to IADLs and relying on pooled samples without stage‐specific analyses.^19^ To our knowledge, only one study has examined cortical atrophy in relation to all three ADL domains, but it was limited to the CU and ADD stages.^20^ Taken together, neuroanatomical findings related to ADL decline remain heterogeneous across domains and disease stages. Temporal, parietal, and frontal cortices are most frequently implicated and are thought to support the cognitive demands of everyday functioning. However, most evidence derives from domain‐restricted and late‐stage studies along the disease continuum, leaving AADLs and stage‐specific neuroanatomical patterns insufficiently characterized.

Another important gap is the underrepresentation of Global South populations, particularly in Latin America (LA). Most available evidence derives from high‐income countries, despite projections indicating that LA will experience one of the highest dementia burdens in the coming decades.^21^ Populations in this region exhibit distinct genetic, cultural, educational, and health‐related characteristics, including lower education attainment and higher multimorbidity rates,^22^, ^23^, ^24^, ^25^ all of which may influence ADL trajectories across the AD continuum. These factors justify the urgent need for population‐specific research on functional decline across the AD continuum in this underrepresented region.

To address these gaps, the present study aimed to characterize ADL performance according to level of complexity across the AD continuum and explore its association with brain atrophy. We leveraged a unique sample comprising a community‐based cohort of Chilean older adults with SCC and MCI and a clinic‐based cohort of individuals with ADD, together representing a clinically defined continuum. Functional ability in BADL, IADL, and AADL was assessed using the Technology‐Activities of Daily Living Questionnaire (T‐ADLQ), an informant‐rated instrument validated in the Chilean population.^26^ Structural magnetic resonance imaging (MRI) was analyzed using voxel‐based morphometry (VBM) to assess regional gray matter (GM) atrophy and identify neuroanatomical correlates of ADL performance.

We hypothesized that, compared to SCC, functional decline in MCI would be restricted to AADL and IADL, whereas ADD would show deficits across all domains. We further expected this decline to be associated with more widespread brain atrophy in ADD than in MCI, involving both domain‐specific and partially overlapping brain regions.

**RESEARCH IN CONTEXT**

1. **Systematic review**: A targeted literature review (e.g., PubMed, Scopus) identified studies examining decline in activities of daily living (ADL)—advanced (AADL), instrumental (IADL), and basic (BADL)—and their neuroanatomical correlates across the Alzheimer's disease (AD) continuum. Prior work shows early functional vulnerability but limited assessment of AADL and scarce evidence from Latin American cohorts. No study has fully integrated ADL complexity with stage‐specific brain atrophy patterns.
2. **Interpretation**: Our findings demonstrate a hierarchical pattern of ADL decline, with early AADL vulnerability followed by progressive IADL and BADL impairment. Stage‐specific gray matter atrophy supports this hierarchy, revealing distinct and partially overlapping neuroanatomical substrates. A multidimensional ADL framework enhances characterization across the AD continuum.
3. **Future directions**: Future studies should incorporate AD biomarkers (positron emission tomography, plasma) to improve classification and include larger and more diverse samples to better characterize functional profiles. Longitudinal and multimodal imaging approaches will refine trajectories of functional decline.

## 2 METHODS

### 2.1 Participants

A total of 138 participants were included in the study: 69 with SCC, 45 with MCI, and 24 with ADD. The groups were matched for age and years of formal education. Individuals with SCC and MCI were recruited from the Chilean GERO (Center for Geroscience, Mental Health and Metabolism) cohort, a community‐dwelling cohort of older adults aged ≥ 70 years.^27^ Participants were identified through a door‐to‐door recruitment strategy in three districts of Santiago, Chile.

Inclusion criteria for the GERO cohort included the presence of cognitive complaints (self‐reported or informant reported), to reduce the likelihood of excluding individuals with MCI who may exhibit limited awareness of cognitive changes; a score of 0 or 0.5 on the Clinical Dementia Rating Scale–Frontotemporal Lobar Degeneration version (CDR‐FTLD);^28^ community residence; and availability of a knowledgeable informant. Self‐reported cognitive complaints were assessed using 11 previously published items and additional questions developed and validated by our group.^29^, ^30^ Informant‐reported cognitive complaints were evaluated using the Eight‐item Informant Interview to Differentiate Aging and Dementia (AD8) questionnaire and other items previously validated by our group.^30^, ^31^ Participants were included if they responded positively to a predefined set of questions (see Material S1 in supporting information). All participants underwent neurological, neuropsychological, and neuroimaging evaluations, and each was accompanied by an informant who provided information about daily functional ability. Dementia was excluded if screening indicated both cognitive impairment, defined as a Mini‐Mental State Examination (MMSE) score < 21, and functional decline, defined as a Pfeffer Functional Activities Questionnaire (PFAQ)^32^ score > 2.

Participants were classified as SCC or MCI according to Diagnostic and Statistical Manual of Mental Disorders Fifth Edition criteria,^33^ based on clinical and neuropsychological assessment. SCC required a cognitive complaint with normal performance on neuropsychological tests and preserved functional independence. In contrast, MCI classification required a subjective cognitive complaint plus objective cognitive impairment on the Montreal Cognitive Assessment (MoCA)^34^ and the Free and Cued Selective Reminding Test (FCSRT),^35^ with functional independence according to the PFAQ.

Participants with ADD were recruited from the Center for Memory and Neuropsychiatry (CMYN) at Hospital del Salvador in Santiago, Chile. They met the National Institute on Aging–Alzheimer's Association (NIA‐AA) criteria for ADD.^2^ Briefly, ADD diagnosis required a clear history of significant episodic memory impairment in the context of preserved behavior and personality, and a global CDR‐FTLD score of ≥ 1. Diagnoses were established by consensus among senior neurologists following a standardized clinical protocol that included informant interviews, laboratory testing, a full neuropsychological assessment, and MRI findings consistent with ADD, as previously described.^36^

Exclusion criteria for all participants included illiteracy, institutionalization, visual or auditory impairments that interfered with assessment, severe mobility limitations, major psychiatric or neurological disorders, or a terminal illness with a life expectancy < 1 year.

### 2.2 ADL assessment

ADL performance in 33 ADLs across seven areas: (1) personal care, (2) home care, (3) work and recreation, (4) shopping and money management, (5) travel, (6) communication, and (7) use of technology was evaluated with the T‐ADLQ completed by a reliable informant.^26^ Each ADL was rated on a four‐point scale (0–3). An additional score of 9 was assigned when the participant had never performed an activity (ND—“never did”) or when the informant lacks sufficient information (DK—“don't know”), allowing adjustment for premorbid functioning and reducing gender and cultural biases. Importantly, T‐ADLQ scores were not used for diagnostic classification.

Based on previous work,^20^, ^37^ the 33 ADL items were classified into three complexity‐based domains: BADL, IADL, and AADL. The T‐ADLQ yields a total impairment score and domain‐specific subscores ranging from 0% to 100%, with higher values indicating greater impairment. Details on scoring procedures and domain composition are presented in Material S2 in supporting information.

### 2.3 Neuropsychological assessment

Participants underwent a complete standardized neuropsychological assessment based on the GERO cohort protocol.^27^ These measures were used to characterize cognitive and neuropsychiatric functioning across groups. Global cognition was assessed with the Addenbrooke's Cognitive Examination (ACE‐III),^38^ and executive function was assessed with the Institute of Cognitive Neurology (INECO) Frontal Screening (IFS),^39^ both validated for the Chilean population. Depressive symptoms were assessed using the Geriatric Depression Scale (GDS).^40^ These same tests were administered to individuals with ADD. A detailed description of the comprehensive neuropsychological protocol is available elsewhere.^27^

### 2.4 MRI acquisition, preprocessing, and analysis

Structural T1‐weighted MRI images were acquired using a T1w magnetization‐prepared rapid gradient echo sequence (repetition time = 1710 ms; echo time = 2.25 ms; field of view = 224 mm; voxel size = 1.0 × 1.0 × 1.0 mm; flip angle = 8°) on a Siemens Skyra 3T scanner equipped with a 20‐channel head coil at the Institute of Neurosurgery Dr. Alfonso Asenjo, Santiago, Chile.

VBM analyses were conducted using Statistical Parametric Mapping (SPM12)^41^ in MATLAB R2022b. Scans were manually aligned to the anterior commissure and reoriented before segmentation into GM, white matter (WM), and cerebrospinal fluid (CSF). A DARTEL template was generated for normalization to the Montreal Neurological Institute (MNI) space, applied to GM images to standardize their spatial representation, and smoothed with an 8‐mm isotropic Gaussian kernel.

The resulting GM maps were analyzed for whole‐brain atrophy using two‐sample *t* tests comparing MCI and ADD groups to SCC. Subsequently, whole‐brain multiple linear regressions were conducted to explore associations between GM atrophy and performance in BADL, IADL, AADL, and total scores. To enhance behavioral variance, increase sample size, and improve statistical power, analyses were conducted for each patient group in tandem with SCC (MCI–SCC; ADD–SCC), rather than separately by group.^42^ Finally, an exclusive mask procedure was applied to contrast the ADL domains between tandem groups (MCI–SCC; ADD–SCC), allowing the identification of domain‐specific patterns of GM atrophy. Subsequently, overlapping regions of GM atrophy associated with the ADL domains were identified using an inclusive masking analysis between these groups.

All analyses were adjusted for total intracranial volume (TIV), estimated using the VBM8 toolbox in SPM12. For group comparisons and regression models involving participants with ADD, whole‐brain family‐wise error (FWE) correction was applied (*p* < 0.05), following standard methodological recommendations for VBM.^41^ In the SCC–MCI analyses, in which subtler effects were expected and no clusters survived FWE correction, voxel‐wise results were reported using an uncorrected threshold of *p* < 0.001 as an exploratory approach. This is consistent with previous VBM studies in early symptomatic stages, in which strict correction may lead to false negatives and obscure meaningful anatomical patterns.^43^ The combined use of *p* < 0.001 uncorrected and *p* < 0.05 FWE thresholds has been reported in SPM‐based neuroimaging studies.^44^ A minimum cluster extent of 50 voxels was applied in all analyses, and anatomical localizations of significant clusters were determined using the Automated Anatomical Labeling (AAL) atlas.^45^

### 2.5 Statistical analysis for sociodemographic, cognitive, and ADL data

Categorical variables were analyzed using chi‐squared (*χ* ^2^) tests. Continuous variables were evaluated with one‐way analysis of variance (ANOVA) followed by Tukey and Games–Howell post hoc tests to account for Type I errors. Non‐parametric variables were assessed using Kruskal–Wallis tests followed by Wilcoxon rank‐sum tests to identify statistically significant differences between groups. A *p* value < 0.05 was considered statistically significant. Analyses were performed using the Statistical Package for the Social Sciences (SPSS) v22 (IBM Corp.) and RStudio v2023.09.1+494.

## 3 RESULTS

### 3.1 Sociodemographic characteristics and inclusion and classification measures across groups

Sociodemographic characteristics, inclusion criteria, and classification measures are presented in Table 1. A significant difference in sex distribution was observed between the SCC and ADD groups. As expected based on the inclusion criteria, groups differed significantly in global cognitive performance on the MMSE. Functional ability on the PFAQ did not differ between SCC and MCI but was significantly more impaired in the ADD group (*p* < 0.05).

**TABLE 1:** Sociodemographic characteristics, inclusion and classification measures, and neuropsychological assessment across groups.

	SCC (1)	MCI (2)	ADD (3)	*𝝌* ^2^ */P* (global)	*p* (post hoc)
**Number of cases** **(*n* = 138)**	69	45	24		
**Sociodemographic characteristics**					
Male/female	15 (21.7%) / 54 (78.3%)	11 (24.4%) / 34 (75.6%)	11 (45.8%) / 13 (54.2%)	0.065^a^	*p*1 = 0.737 (1–2) ***p*2 = 0.023 (1–3)** *p*3 = 0.069 (2–3)
Age (years)	74.0 (72.0–77.0)	75.0 (73.0–78.5)	76.5 (71.2–81.7)	0.198^b^	*p*1 = 0.145 (1–2) *p*2 = 0.154 (1–3) *p*3 = 0.613 (2–3)
Education (years)	10 (6–13)	8 (6–12)	12 (8–16)	0.294^b^	*p*1 = 0.450 (1–2) *p*2 = 0.317 (1–3) *p*3 = 0.108 (2–3)
**Inclusion measures**					
MMSE	29.0 (27.75–29.00)	27.0 (25.00–27.75)	22.0 (18.25–25.00)	**<0.001** ^b^	***p*1 < 0.001 (1–2)** ***p*2 < 0.001 (1–3)** ***p*3 = 0.002 (2–3)**
PFAQ	0.00 (0.00–1.00)	0.00 (0.00–1.00)	8.00 (6.00–14.00)	**<0.001** ^b^	*p*1 = 1.000 (1–2) ***p*2 < 0.001 (1–3)** ***p*3 < 0.001 (2–3)**
**Classification measures**					
MoCA	25.0 (23.0–27.0)	19.0 (16.0–20.0)	16.0 (13.2–18.0)	**<0.001** ^b^	***p*1 < 0.001 (1–2)** ***p*2 < 0.001 (1–3)** *p*3 = 0.196 (2–3)
FR–FCSRT	34.68 ± 3.96 (27–45)	28.73 ± 5.15 (19–44)	13.45 ± 6.79 (1–24)	**<0.001** ^c^	***p*1 < 0.001 (1–2)** ***p*2 < 0.001 (1–3)** ***p*3 < 0.001 (2–3)**
TR–FCSRT	48.0 (48.0–48.0)	47.5 (46.0–48.0)	38.0 (31.2–44.2)	**<0.001** ^b^	***p*1 = 0.002 (1–2)** ***p*2 < 0.001 (1–3)** ***p*3 < 0.001 (2–3)**
Neuropsychological assessment					
ACE‐III	86.72 ± 6.57 (72–100)	73.44 ± 10.98 (43–95)	61.62 ± 12.83 (37–82)	**<0.001** ^c^	***p*1 < 0.001 (1–2)** ***p*2 < 0.001 (1–3)** ***p*3 = 0.001 (2–3)**
IFS	18.97 ± 4.16 (7–26)	14.54 ± 5.35 (3–25.5)	11.31 ± 4.71 (3–20)	**<0.001** ^d^	***p*1 < 0.001 (1–2)** ***p*2 < 0.001 (1–3)** ***p*3 = 0.020 (2–3)**
GDS	2.00 (0.00–4.00)	3.00 (1.00–4.00)	4.00 (0.25–7.75)	0.234^b^	*p*1 = 0.313 (1–2) *p*2 = 0.136 (1–3) *p*3 = 0.263 (2–3)

*Notes*: Data are presented as median (Q1–Q3) and mean ± standard deviation (minimum–maximum), except for sex, which is expressed as a percentage. *p*1: SCC versus MCI; *p*2: SCC versus ADD; *p*3: MCI versus ADD. Significance level: *p* < 0.05.

Abbreviations: ACE‐III, Addenbrooke's Cognitive Examination III; ADD, Alzheimer's disease dementia; ANOVA, analysis of variance; FR‐FCSRT, Free Recall, Free and Cued Selective Reminding Test; GDS, Geriatric Depression Scale; IFS, INECO Frontal Screening; MCI, mild cognitive impairment; MMSE, Mini‐Mental State Examination; MoCA, Montreal Cognitive Assessment; PFAQ, Pfeffer Functional Activities Questionnaire; SCC, subjective cognitive complaints; TR‐FCSRT, Total Recall, Free and Cued Selective Reminding Test.

^a^ Chi‐squared test.

^b^ Kruskal–Wallis test (post hoc: Wilcoxon rank‐sum).

^c^ Welch one‐way ANOVA (post hoc: Games–Howell).

^d^ One‐way ANOVA (post hoc: Tukey).

Consistent with the classification measures, the SCC group performed significantly better than both the MCI and ADD groups on the MoCA and episodic memory measures from the FCSRT (Free Recall [FR] and Total Recall [TR]). The MCI group also scored significantly higher than the ADD group on episodic memory measures, but not on the MoCA (*p* < 0.05; Table 1).

### 3.2 Neuropsychological assessment across groups

Neuropsychological measures used to characterize cognitive and neuropsychiatric performance across groups are presented in Table 1. Significant differences were observed across groups on the ACE‐III, with progressively lower scores from SCC to MCI and ADD (*p* < 0.05). Executive function, assessed with the IFS, also differed significantly across groups (*p* < 0.05), with progressively lower scores across successive clinical stages. Participants with SCC performed within the expected normative range for Chilean older adults, whereas the MCI and ADD groups showed increasing levels of cognitive impairment. No differences were observed in depressive symptoms measured by the GDS.

### 3.3 Total and domain‐specific functional decline across groups

**FIGURE 1:**
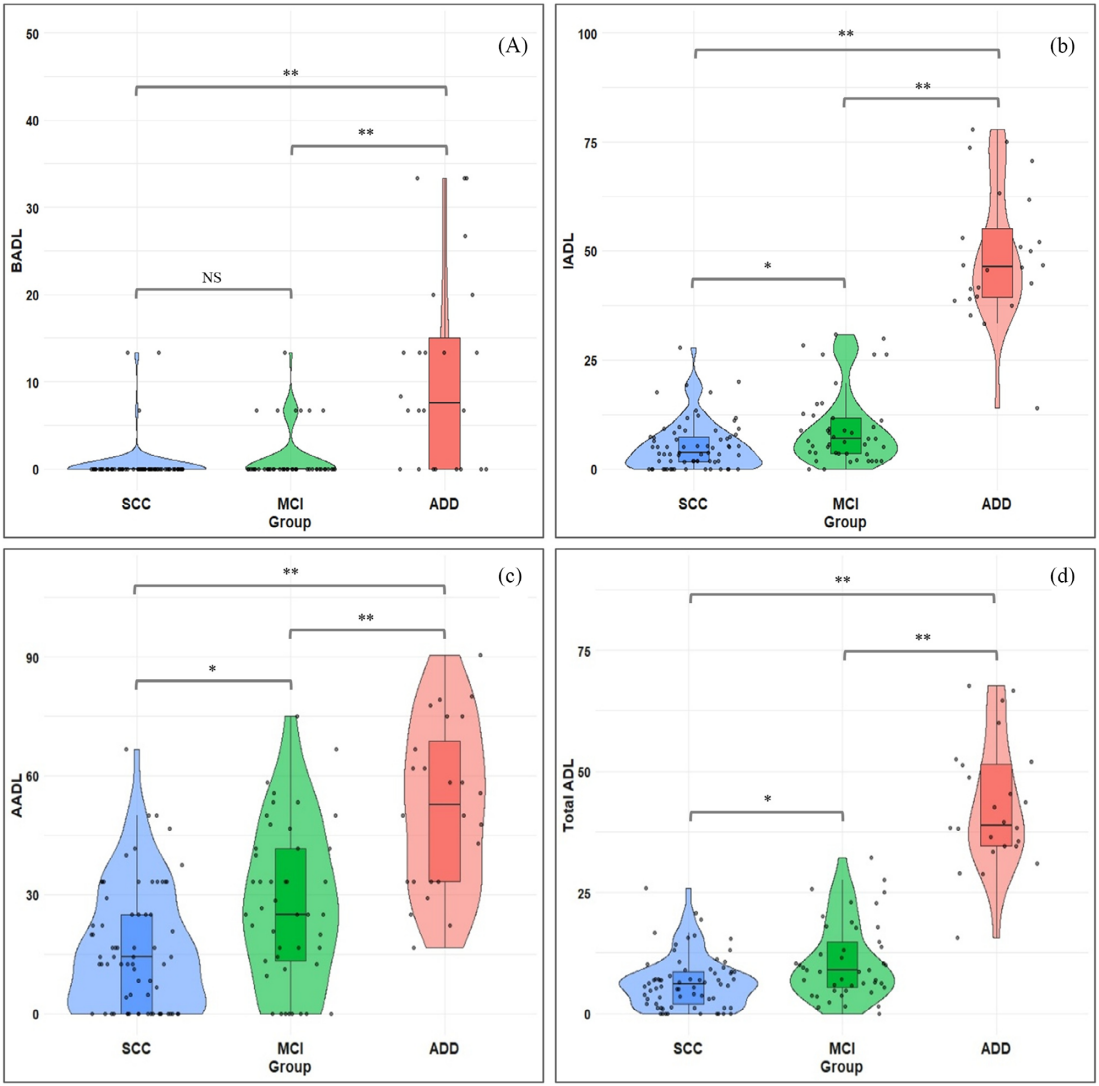
Total and domain‐specific T‐ADLQ scores across groups. A, BADL, (B) IADL, (C) AADL, (D) total ADL** *p* < 0.001; * *p* < 0.05. *n* = 138 (69 = SCC; 45 = MCI; 24 = ADD). AADL, advanced activities of daily living; ADD, Alzheimer's disease dementia; ADL, activities of daily living; BADL, basic activities of daily living; IADL, instrumental activities of daily living; MCI, mild cognitive impairment; NS, non‐significant differences; SCC, subjective cognitive complaints.

The ADD group exhibited significantly greater functional decline compared to both the SCC and the MCI groups on the T‐ADLQ total score and on all three domains—AADL, IADL, and BADL (*p* < 0.05; Table 2; Figure 1A–B). SCC and MCI differed significantly on the AADL subscore (Figure 1C), while differences in the IADL subscore and total T‐ADLQ score were only marginally significant (*p* = 0.044 and *p* = 0.033, respectively; Table 2; Figures 1B and 1D). No significant differences were detected in the BADL subscore (Figure 1A).

**TABLE 2:** Total and domain‐specific ADL scores across clinical groups.

	SCC (1)	MCI (2)	ADD (3)	*𝝌* ^2^ */P* (global)	*p* (post hoc)
**Number of cases (*n* = 138)**	69	45	24		
**ADL variables**					
BADL	0.00 (0.00–0.00)	0.00 (0.0–0.00)	7.50 (0.00–18.33)	**<0.001** ^b^	*p*1 = 0.527 (1–2) ***p*2 < 0.001 (1–3)** ***p*3 < 0.001 (2–3)**
IADL	3.70 (1.63–7.67)	7.02 (3.51–11.97)	46.41 (39.06–59.48)	**<0.001** ^b^	***p*1 = 0.044 (1–2)** ***p*2 < 0.001 (1–3)** ***p*3 < 0.001 (2–3)**
AADL	14.29 (0.00–27.08)	25.00 (12.91–41.67)	52.77 (33.33–72.91)	**<0.001** ^b^	***p*1 = 0.013 (1–2)** ***p*2 < 0.001 (1–3)** ***p*3 < 0.001 (2–3)**
Total ADL score	6.06 (2.02–8.80)	8.89 (5.04–16.26)	38.88 (34.54–51.82)	**<0.001** ^b^	***p*1 = 0.033 (1–2)** ***p*2 < 0.001 (1–3)** ***p*3 < 0.001 (2–3)**

Abbreviations: AADL, advanced activities of daily living; ADD, Alzheimer's disease dementia; ADL, activities of daily living; ANOVA, analysis of variance; BADL, basic activities of daily living; IADL, instrumental activities of daily living; MCI, mild cognitive impairment; SCC, subjective cognitive complaints.

*Notes*: Data are presented as median (Q1–Q3) and mean ± standard deviation (minimum–maximum), except for sex, which is expressed as a percentage. *p*1: SCC versus MCI; *p*2: SCC versus ADD; *p*3: MCI versus ADD. Significance level: *p* < 0.05.

^a^ Chi‐squared test.

^b^ Kruskal–Wallis test (post hoc: Wilcoxon rank sum).

^c^ Welch one‐way ANOVA (post hoc: Games–Howell).

^d^ One‐way ANOVA (post hoc: Tukey).

Given the sex imbalance observed across groups, a sensitivity analysis using a diagnosis‐by‐sex factorial model was performed to assess the potential impact on ADL performance. This analysis revealed no significant main effect of sex and no diagnosis‐by‐sex interaction (Table S1 in supporting information), indicating that the group differences reported above were not driven by sex‐related confounding.

Within the SCC group, AADL performance showed marked interindividual variability, with some participants exhibiting marked impairments (Figure 1C). The ADD group scored significantly worse than SCC and MCI across all seven functional areas (*p* < 0.05; Table S2 in supporting information). Comparisons between SCC and MCI revealed a marginal difference in the work and recreation area (*p* = 0.038) and a significant difference in the technology use area (*p* = 0.002), both falling within the AADL and IADL domains (Material S2 and Table S2).

In addition, the distribution of informant types was similar across groups (*χ* ^2^ test, *p* > 0.05; Table S3 in supporting information).

### 3.4 GM atrophy across groups

VBM analyses revealed that individuals with MCI compared to those with SCC exhibited significantly greater GM atrophy in frontal and temporal regions, particularly in the left inferior frontal gyrus (opercular part), left supplementary motor area, and bilateral medial temporal gyri (*p* < 0.001, uncorrected; Figure S1 and Table S4 in supporting information).

In contrast, the comparison between the SCC and ADD groups revealed a more widespread pattern of GM atrophy in the ADD group. Significant reductions were observed in the bilateral hippocampi; left inferior temporal gyrus; bilateral medial temporal gyri; left fusiform gyrus; bilateral anterior orbital gyri; left angular gyrus; right inferior parietal gyrus; and left inferior occipital gyrus (*p* < 0.05, FWE corrected; Figure S2 and Table S5 in supporting information).

The comparison between the MCI and ADD groups further revealed greater GM atrophy in the ADD group, predominantly involving medial temporal and parietal regions. Significant reductions were observed in the bilateral hippocampi, right inferior parietal gyrus, and right middle temporal gyrus (*p* < 0.05, FWE corrected; Figure S3 and Table S6 in supporting information).

### 3.5 Associations between GM volume and total/domain‐specific ADL performance

Higher total T‐ADLQ scores were associated with reduced GM volume in temporal, frontal, and occipital regions in the MCI–SCC tandem group (*p* < 0.001, uncorrected; Figure S4 and Table S7 in supporting information). A similar but more widespread pattern, including parietal and subcortical regions, was observed in the ADD–SCC tandem group (*p* < 0.05, FWE corrected; Figure S5 and Table S8 in supporting information).

**FIGURE 2:**
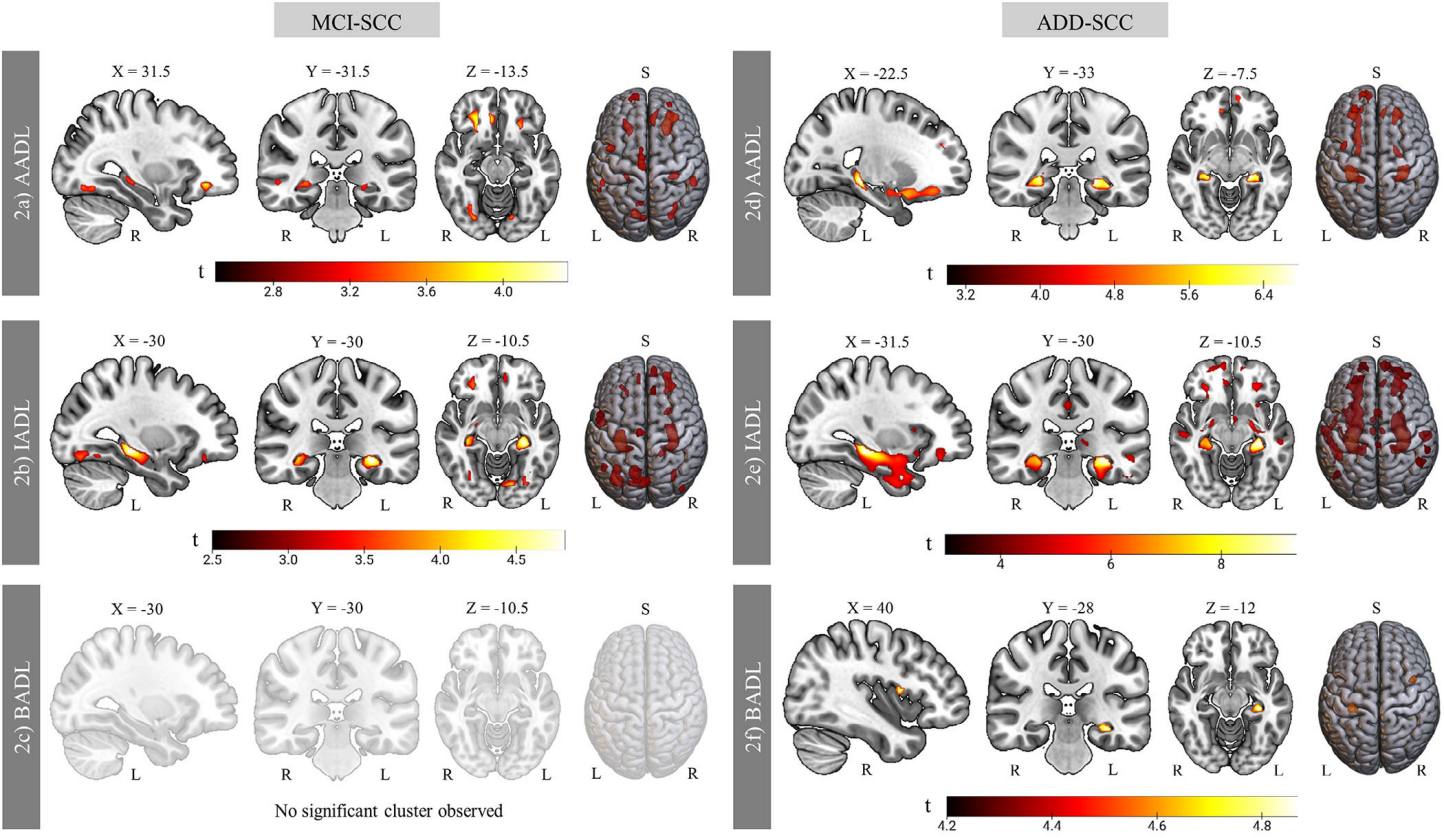
Association between gray matter volume and ADL domain performance. Coordinates are reported in Montreal Neurologic Institute space (*x*, *y*, *z*). Color bars indicate *t* values. Clusters for the MCI group are shown at *p* < 0.001 (uncorrected), and clusters for the ADD group at *p* < 0.05 (family‐wise error corrected). *n* = 138 (69 = SCC; 45 = MCI; 24 = ADD). In the MCI‐SCC group, reduced GM volume was associated with AADL (2a) and IADL (2b), but not BADL (2c). In the ADD‐SCC group, reduced GM volume was associated with AADL (2d), IADL (2e), and BADL (2f). AADL, advanced activities of daily living; ADD, Alzheimer's disease dementia; ADL, activities of daily living; BADL, basic activities of daily living; IADL, instrumental activities of daily living; L, left; MCI, mild cognitive impairment; R, right; S, superior view; SCC, subjective cognitive complaints.

Reduced AADL performance in the MCI–SCC group was associated with lower GM volume in temporal, frontal, occipital, and insular regions (*p* < 0.001, uncorrected; Figure 2A; Table S9 in supporting information). In the ADD–SCC group, associations were more focal, mainly involving frontal, temporal, and insular regions (*p* < 0.05, FWE corrected; Figure 2D; Table S10 in supporting information).

Similarly to AADLs, reduced IADL performance in the MCI–SCC group was associated with GM reductions in temporal, frontal, and occipital regions, with a broader distribution than that observed for AADLs (*p* < 0.001, uncorrected; Figure 2B; Table S11 in supporting information). In the ADD–SCC group, IADL performance was associated with an even more extensive pattern, including temporal, frontal, parietal, insular, and subcortical regions (*p* < 0.05, FWE corrected; Figure 2E; Table S12 in supporting information).

For BADL, no significant associations were observed in the MCI–SCC group (Figure 2C). In contrast, in the ADD–SCC group, decreased BADL performance was associated with reduced GM volume in the left hippocampus and right insula (*p* < 0.05, FWE corrected; Figure 2F; Table S13 in supporting information).

In the MCI–SCC group, reduced technology‐related functional performance was associated with lower GM volume in the bilateral lingual gyri and the right fusiform gyrus (*p* < 0.001, uncorrected; Figure S6 and Table S14 in supporting information). No significant associations were observed for technology use in the ADD–SCC group.

### 3.6 Exclusive and overlapping patterns between tandem groups by ADL domain

As several significant clusters extended across multiple cortical and subcortical regions, portions of the same anatomical areas appeared in more than one regression model. Below, we detail domain‐specific and shared regions.

**FIGURE 3:**
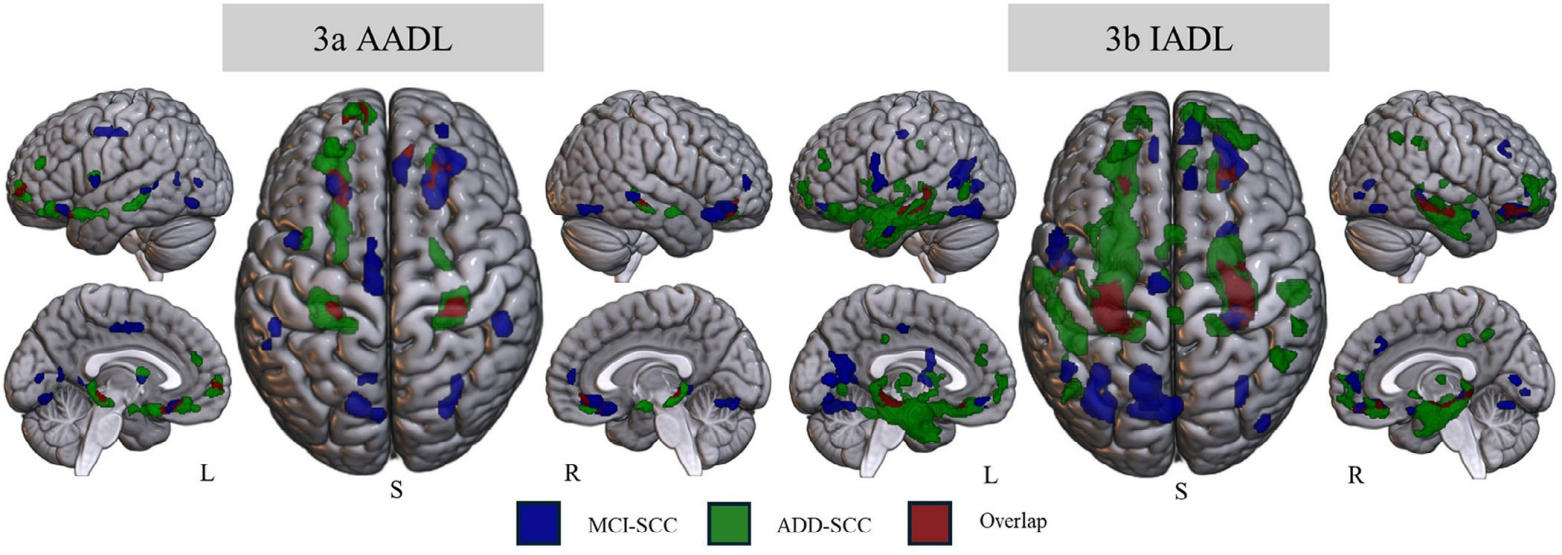
Exclusive and overlapping patterns between tandem groups by ADL domain. Clusters for the MCI group are shown at *p* < 0.001 (uncorrected). Clusters for the ADD group and overlap regions are shown at *p* < 0.05 (family‐wise error corrected). *n* = 138 (69 = SCC; 45 = MCI; 24 = ADD). AADL (3a) and IADL (3b) show group‐specific and shared associations with GM volume: blue indicates MCI‐SCC‐exclusive associations, green indicates ADD‐SCC‐exclusive associations, and red indicates overlapping regions between groups. AADL, advanced activities of daily living; ADD, Alzheimer's disease dementia; ADL, activities of daily living; BADL, basic activities of daily living; IADL, instrumental activities of daily living; L, left; MCI, mild cognitive impairment; R, right; S, superior view; SCC, subjective cognitive complaints.

In the MCI–SCC group, AADL performance was exclusively associated with reduced GM volume in portions of frontal, temporal, occipital, and insular regions, including the right superior frontal gyrus (medial orbital and dorsolateral parts), right medial orbital gyrus, left posterior orbital gyrus, left supplementary motor area, bilateral medial temporal gyri, right fusiform gyrus, left lingual gyrus, left calcarine cortex, and left insula (*p* < 0.001, uncorrected; Figure 3A; Table S15 in supporting information).

In the ADD–SCC group, exclusive associations for AADL performance involved reduced GM volume in portions of frontal, temporal, and insular regions, including bilateral anterior orbital gyri, left superior frontal gyrus (dorsolateral and medial orbital parts), left medial frontal gyrus, left posterior orbital gyrus, right hippocampus, bilateral parahippocampal gyri, and left insula (*p* < 0.05, FWE corrected; Figure 3A; Table S16 in supporting information).

Overlapping regions for AADL performance in both groups involved GM reductions in portions of the bilateral anterior orbital gyri, right superior frontal gyrus (medial orbital part), left superior frontal gyrus (dorsolateral part), left parahippocampal gyrus, and right hippocampus (*p* < 0.05, FWE corrected; Figure 3A; Table S17 in supporting information).

In the MCI–SCC group, exclusive associations for IADL performance involved reduced GM volume in portions of frontal, temporal, occipital, and insular regions, including bilateral fusiform gyri, right hippocampus, left medial and inferior temporal gyri, left precentral gyrus, right anterior orbital gyrus, right superior frontal gyrus (medial part), left supplementary motor area, right medial cingulate and paracingulate gyri, left superior frontal gyrus (medial orbital part), left calcarine cortex, left lingual gyrus, right inferior occipital gyrus, and right insula (*p* < 0.001, uncorrected; Figure 3B; Table S18 in supporting information).

In the ADD–SCC group, exclusive associations for IADL performance involved reduced GM volume in a broader network encompassing portions of frontal, temporal, parietal, insular, and subcortical regions, including the left parahippocampal gyrus, right hippocampus, left medial and inferior temporal gyri, bilateral superior temporal gyri, bilateral superior frontal gyri (dorsolateral parts), right anterior orbital gyrus, right anterior and medial cingulate gyri, right superior frontal gyrus (medial orbital part), right inferior parietal and supramarginal gyri, left insula, and bilateral mediodorsal magnocellular thalamic nuclei (*p* < 0.05, FWE corrected; Figure 3B; Table S19 in supporting information).

Overlapping regions associated with IADL performance in both groups included GM reductions in portions of the bilateral hippocampi, bilateral anterior orbital gyri, and the left inferior temporal gyrus (*p* < 0.05, FWE corrected; Figure 3B; Table S20 in supporting information).

## 4 DISCUSSION

This study examined functional performance in three hierarchically complex ADL domains—BADL, IADL, and AADL—across the AD continuum from SCC to MCI and ADD. By integrating clinical stage, ADL complexity, and brain–behavior relationships, we provide a multidimensional characterization. The inclusion of adults > 70 years from an LA cohort broadens evidence beyond high‐income samples, supporting a context‐sensitive understanding of functional decline.

Functional decline varied across the continuum in severity and ADL complexity. AADL and IADL were affected in earlier stages, whereas BADL impairment emerged only in advanced stages. Brain–behavior analyses revealed both exclusive and overlapping GM atrophy patterns across ADL complexity. In MCI–SCC, AADL decline was associated with more restricted atrophy patterns, whereas in ADD–SCC, impairment across domains corresponded to progressively widespread atrophy from AADL to BADL. These findings are consistent with an anatomically ordered pattern of functional differences—from complex to basic—across clinical stages.

### 4.1 Hierarchical patterns of ADL decline

Behavioral results from the T‐ADLQ revealed that, compared to SCC, individuals with MCI showed greater AADL impairment and mild IADL deficits, while BADL remained preserved. In ADD, impairment was observed across all domains. In SCC, AADL ranged widely (0%–60%), with narrower IADL variability (0%–25%), consistent with reports of subtle limitations in CU older adults.^26^, ^37^, ^46^, ^47^ This variability likely reflects non‐cognitive determinants such as education, multimorbidity, age‐related changes, and gender roles.^46^

As AADL rely on higher‐order processes, they decline early, even at preclinical stages and before IADL,^9^, ^48^, ^49^ explaining the variability observed in our SCC group. The significant difference in AADL between SCC and MCI supports progressive decline in complex ADL.^49^ Although common in MCI, AADL impairments typically do not compromise independence.^10^ Both groups showed IADL limitations with marginal differences, suggesting subtle inefficiencies. Moreover, IADL limitations increase dementia risk and become more evident during the transition from MCI to dementia.^50^

BADL remained preserved in SCC and MCI and declined only in ADD, consistent with their later involvement.^9^, ^49^, ^51^ BADL impairment in ADD was mild (median = 7.5%), aligning with evidence that BADL deteriorates once global cognitive impairment becomes pronounced.^20^, ^51^

These findings, together with longitudinal evidence,^7^ support a progressive and hierarchical functional decline that parallels cognitive deterioration and highlight the importance of assessing ADL across levels of complexity.

### 4.2 Neuroanatomical correlates of functional decline in ADLs

GM atrophy patterns associated with each ADL domain reflected the hierarchical decline. To our knowledge, this is the first study to explore AADL–atrophy relationships in MCI. In MCI–SCC, AADL impairment showed exclusive associations with widespread frontal, temporal, occipital, and insular regions, whereas in ADD–SCC, associations were more concentrated in frontal, temporal, and insular regions. Overlap analyses identified shared regions, including the bilateral anterior orbital gyri, superior frontal gyrus, left parahippocampal gyrus, and right hippocampus.

Temporal involvement in ADD–SCC aligns with the only previous study linking AADL impairment to GM atrophy in ADD.^20^ Our findings extend this by identifying additional frontal and insular associations, supporting AADL reliance on distributed cognitive networks.^49^ Differences from previously reported regions, such as the left lingual gyrus or intracalcarine cortex,^20^ may reflect methodological or sample variations.

Regions shared across MCI–SCC and ADD–SCC suggest early frontotemporal and medial temporal involvement typical of AD.^52^ These regions may represent common structural vulnerabilities that support executive control, episodic memory, social cognition, and behavioral regulation,^20^, ^53^ which are essential for AADL performance.

For IADL, exclusive associations in MCI–SCC involved frontal, temporal, occipital, and insular regions, whereas in ADD–SCC they expanded to frontal, temporal, parietal, insular, and subcortical regions. Overlap included the bilateral hippocampi, the anterior orbital gyri, and the left inferior temporal gyrus. Findings align with evidence linking IADL decline in MCI to medial temporal atrophy, including the hippocampus, entorhinal cortex, and amygdala.^15^ Financial capacity, a complex IADL, has been associated with frontal and temporal atrophy.^54^ Although occipital involvement is less reported, our results suggest these associations are consistent with occipital roles in visuospatial processing, navigation, and orientation,^55^ which are relevant to IADL.^56^ In MCI–SCC, technology‐related performance was associated with occipito‐temporal regions, including the lingual and fusiform gyri, consistent with visual and symbolic processing demands of technology use.^57^

The GM atrophy pattern associated with IADL in ADD–SCC aligns with widespread degeneration reported in ADD and is reflected in global atrophy indices, including the ventricle–brain ratio.^18^, ^20^, ^53^, ^58^ Studies of financial capacity in ADD implicate frontal and parietal atrophy without hippocampal involvement, underscoring executive and perceptual–motor integration networks.^59^ Evidence from mixed cohorts (CU, SCC, MCI, ADD) reports temporal, frontal, parietal, and occasional cerebellar involvement.^19^, ^60^, ^61^ However, our results extend this evidence by delineating stage‐specific IADL–atrophy relationships.

Regions shared across MCI–SCC and ADD–SCC suggest a common structural core underlying IADL impairment. These regions align with large‐scale brain networks, including frontoparietal control, default mode, dorsal attention, visual, and salience networks, which support executive, mnemonic, visuospatial, attentional, and behavioral regulation.^62^, ^63^ Hippocampal involvement, commonly observed in MCI and ADD,^52^ further supports underlying AD pathology in our MCI group.

An inverse pattern emerged across disease stages. In MCI–SCC, AADL showed broader associations, likely reflecting subtle cognitive changes^64^ that elicit compensatory mechanisms through distributed networks.^65^ Alternatively, the more restricted AADL associations in ADD–SCC may reflect a floor effect,^66^ with reduced interindividual variability and limited detection of significant associations. IADL showed the opposite pattern: in MCI–SCC, limited associations align with the mild functional impairment, whereas in ADD–SCC, broader associations parallel the greater IADL decline and correspond to widespread neurodegeneration and network‐level propagation typical of AD,^67^ requiring further validation.

Regarding BADL, no associations emerged in MCI–SCC, consistent with preserved BADL at this stage.^51^ In ADD–SCC, BADL decline involved the left hippocampus and right insula, consistent with reports also implicating frontal, parietal, and basal ganglia regions.^20^, ^68^

As a salience‐network node,^62^ the right insula supports interoception, sensorimotor integration, and autonomic regulation,^69^ which are essential for basic self‐care. Although evidence linking insular atrophy to impairment is limited, left‐insular atrophy has been associated with IADL deficits in frontotemporal dementia,^70^ consistent with left‐insular involvement in AADL and IADL in our sample. Right insula integrity has also been associated with greater awareness of memory deficits,^71^ suggesting a role in self‐monitoring. These findings underscore a central role of the right insula.

### 4.3 Clinical implications

These findings highlight the value of assessing ADL complexity to characterize functional phenotype across the AD continuum. Early vulnerability of AADL, reflected in SCC variability and early MCI decline, supports their role as sensitive markers of initial impairment, whereas the slower decline of IADL and BADL underscores their utility in later stages. Integrating this ADL framework with biomarkers and multimodal imaging may improve patient stratification and diagnostic strategies.

### 4.4 Strengths and limitations

This study includes individuals across the AD continuum, enabling integrated analysis of functional changes and their neuroanatomical correlates. The T‐ADLQ, which captures hierarchical ADL complexity, combined with VBM, provided a multidimensional, culturally sensitive assessment.

This study has several limitations. The absence of a control group without cognitive complaints may reduce the accuracy of group differentiation. MCI classification required SCCs reported by the participant or an informant. Although consistent with diagnostic frameworks, this criterion may include individuals with preserved awareness and limit generalizability to those with emerging anosognosia in MCI.^72^ Informant reports may reduce exclusion, but some selection bias cannot be excluded. Additionally, diagnoses across the AD continuum relied solely on clinical criteria without biomarker confirmation; however, evidence supporting their diagnostic validity in LA remains limited.^21^, ^73^

Given the cross‐sectional design, hierarchical functional decline is inferred from between‐group differences; longitudinal studies are needed to validate these trajectories.

Heterogeneity within the SCC group may have influenced effect sizes or anatomical patterns and attenuated between‐group comparisons. Nonetheless, SCC serves as a proxy for CU given preserved neuropsychological performance,^74^ and structural analyses showed that MCI and ADD exhibit GM atrophy in typical AD‐related regions relative to SCC, supporting its use as a reference group (Figures S1–S3; Tables S4–S6).

Additionally, there is an overrepresentation of women, which is common in cognitive complaints cohorts.^75^ No statistical adjustment for sex was applied in neuroimaging analyses. Although the T‐ADLQ minimizes gender‐related role bias by excluding non‐performed activities,^26^ and behavioral sensitivity analyses showed no effect of sex on ADL performance, the imbalance in sex distribution between SCC and ADD should be considered when interpreting neuroimaging findings. The smaller ADD sample may also reduce statistical power.

Although informant type did not differ across groups (Table S3), informant‐based measures remain susceptible to bias. Informant characteristics, including caregiver burden and culturally shaped expectations about aging and functional performance,^6^ may influence ratings and should be considered.

Finally, comparison and regression results in MCI–SCC did not survive FWE correction and were therefore reported at *p* < 0.001 uncorrected. Such thresholds are often used when effects are subtle but remain exploratory, as uncorrected voxel‐wise thresholds increase sensitivity but also increase the likelihood of false positives.^76^

## 5 CONCLUSION

This study demonstrates that stratifying ADL by complexity provides a sensitive and clinically meaningful framework to characterize functional changes across the AD continuum. Integrating functional performance with structural neuroimaging identified stage‐dependent and partially overlapping anatomical patterns, consistent with earlier loss of complex activities. These findings support multidimensional ADL assessments in clinical and research settings, with the inclusion of an LA cohort underscoring relevance in diverse populations. Future studies combining functional measures with biomarkers may improve patient stratification and early detection. Longitudinal studies will be essential to validate these stage‐specific patterns and clarify functional decline trajectories.

## AUTHOR CONTRIBUTIONS

**Fernando Henriquez**: data curation, methodology, formal analysis, writing–original draft. **Patricio Riquelme**: methodology, writing–review & editing. **Gonzalo Forno**: writing–review & editing. **Joaquin Migeot**: methodology, writing–review & editing. **Rodrigo Henriquez**: writing–review & editing. **Patricia Lillo**: writing–review & editing. **Daniela Thumala‐Dockendorff**: writing–review & editing. **Cecilia Okuma**: writing–review & editing. **Cecilia Gonzalez‐Campo**: methodology, validation, writing–review & editing. **Michael Hornberg**: methodology, validation, writing–review & editing. **Francisco Aboitiz**: methodology, writing–review & editing. **Andrea Slachevsky**: conceptualization, methodology, writing–review & editing, supervision, project administration, funding acquisition. All authors read and approved the final manuscript.

## CONFLICT OF INTEREST STATEMENT

The authors declare that the research was conducted in the absence of any commercial or financial relationships that could be construed as a potential conflict of interest. Author disclosures are available in the Supporting Information

## DISCLAIMER

The content is solely the responsibility of the authors and does not necessarily represent the official views of the National Institute on Aging, the Alzheimer's Association, the Rainwater Charitable Foundation, or the Bluefield Project to Cure Frontotemporal Dementia. Study data were partially collected and managed using REDCap electronic data capture tools hosted at the Faculty of Medicine, Universidad de Chile.

## CONSENT STATEMENT

All participants provided written informed consent in accordance with the Declaration of Helsinki. The Servicio de Salud Metropolitano Oriente Ethics Committee, Santiago, Chile, approved the GERO Cohort project. The Chilean National Agency of Research (ANID, FONDAP 15150012) funds the project.

## Supporting information



Supporting Information: alz71445‐sup‐0001‐SuppMat.docx

Supporting Information: alz71445‐sup‐0002‐SuppMat.pdf
