# Supplementary material for: Activities of daily living and their neural correlates across the Alzheimer's disease continuum: Evidence from a Latin American cohort
Source: Alzheimers Dement. 2026 Apr 29;22(5):e71445. doi: 10.1002/alz.71445 (PMC13128346; doi:10.1002/alz.71445)
Supplement: Supplementary file 1 — Supporting Information: alz71445‐sup‐0001‐SuppMat.docx [file ALZ-22-e71445-s001.docx]

**Supplementary material**

**Supplementary material 1:** Participant- and informant -reported cognitive complaints

Cognitive Complaint Scale – Participant Questionnaire

| No. | Question |
| --- | --- |
| 1 | How is your memory compared to the way it was 10 years ago?   - Better - Same - Worse |
| 2 | Do you remember things less well than you did a year ago?   - Yes - No - I do not know |
| 3 | How is your ability to – accurately remember the names of close friends and relatives?   - Very good - Good - Bad - Very bad |
| If the response to question 3 was Bad or Very Bad →Positive cognitive complaint (participant). If not →Continue | |
| 4 | Do you believe your memory has become worse?   - Yes - No - I do not know |
| 5 | Do you usually forget where things are?   - Yes - No - I do not know |
| 6 | Do you have difficulties finding the right words during a conversation?   - Yes - No - I do not know |
| 7 | Do you remember accurately appointments?   - Very good - Good - Bad - Very bad |
| 8 | Do you have trouble remembering things that have recently happened?   - Yes - No - I do not know |
| 9 | Do you ever forget what you were told yesterday or the day before?   - Yes - No - I do not know |
| If questions 8 and 9 were answered “Yes” → Positive Cognitive Complaint (participant).  If not → Negative Cognitive Complaint (participant). | |
| 10 | Do you ever start doing something and forget what you were doing?   - Yes - No - I do not know |
| 11 | Do you ever go to a room to look for something and forget what you came for?   - Yes - No - I do not know |

Cognitive Complaint Scale – Reliable Informant Questionnaire

Informant initial evaluation. Inclusion questions

| No. | Question | Yes | No |
| --- | --- | --- | --- |
| 1 | Have you, or has someone close to you, noticed significant changes in the memory of your relative that cause you concern? |  |  |
| 2 | Are these memory changes concerning enough for you to consider seeking professional evaluation or consultation? |  |  |
| 3 | Have you sought, or has anyone suggested that you seek professional evaluation for this problem? |  |  |
| If you have not sought evaluation, please indicate the reason(s) *(select all that apply):*  *a) You did not consider it necessary and therefore did nothing.*  *b) You did not consider it necessary and used home remedies.*  *c) You preferred to consult a pharmacist for medication related to the health problem.*  *d) You preferred to consult a specialist in alternative medicine (e.g., vibrational medicine, biomagnetism, reiki, iridology, Bach flower therapy, Eastern medicine, Indigenous medicine, naturopathy, or homeopathy).*  *e) You considered seeking consultation but did not have time.*  *f) You considered seeking consultation but did not have sufficient financial resources.*  *g) You considered seeking consultation but found it difficult to access the healthcare facility.*  *h) You requested an appointment but were unable to obtain one.*  *i) You obtained an appointment, but it has not yet taken place.*  *j) obtained an appointment but did not attend*  *k) Do_not know /_Not applicable* | | | |

AD8. Below are a series of situations that refer to the person you are reporting on. In your case, Mr./Ms. [participant's name]. Please answer “Yes” IF YOU CONSIDER THAT THERE HAS BEEN ANY CHANGE in the aspect indicated over the last few years, due to cognitive problems (thinking, memory, etc.)

| No. | Situation | Yes | No | I do not know / Not applicable |
| --- | --- | --- | --- | --- |
| 1 | Problems making judgments and decisions (being deceived, making poor financial decisions, giving inappropriate gifts). |  |  |  |
| 2 | Loss of interest in hobbies and activities (e.g., has stopped doing activities that they used to enjoy). |  |  |  |
| 3 | Repeats questions, comments, or things that are said. |  |  |  |
| 4 | Difficulty learning how to use tools, appliances, or devices (e.g., video or DVD player, computer, microwave, remote control, cell phone, or cordless phone). * |  |  |  |
| 5 | Forgets the correct month or year. * |  |  |  |
| 6 | Difficulty managing complicated financial matters (e.g., making payments, paying taxes, bills, receipts, etc.). |  |  |  |
| 7 | Difficulty remembering appointments and things they must do. |  |  |  |
| 8 | Problems with reasoning and/or memory are daily occurrences, not occasional ones. |  |  |  |

Informant ABCD-Q

| No. | Item | Strongly disagree | Disagree | Agree | Strongly agree |
| --- | --- | --- | --- | --- | --- |
| 9 | His/Her memory or concentration difficulties make it harder for you to live independently. |  |  |  |  |
| 10 | He/She is more indifferent to the feelings or problems of others. |  |  |  |  |
| 11 | He/She is less aware of when your behavior bothers or annoys others. |  |  |  |  |
| 12 | His/Her comments or behavior have become more inappropriate (e.g., poor manners, use of profanity, or other socially inappropriate actions). |  |  |  |  |
| 13 | He/She finds it more difficult to organize and express his/her ideas. |  |  |  |  |
| 14 | He/She finds it harder to make appropriate decisions |  |  |  |  |
| 15 | He/She finds it harder to maintain concentration on what he/she is doing. |  |  |  |  |
| 16 | He/She is confused about the order in which events occurred. |  |  |  |  |
| 17 | He/She has become more forgetful. * |  |  |  |  |
| 18 | He/She tends to repeat himself/herself more often. |  |  |  |  |
| 19 | He/She has greater difficulty remembering conversations that took place a few days ago |  |  |  |  |

If any of the questions in bold was answered “Yes” (questions 4 or 5) or Agree / Strongly Agree (question 17) →Positive cognitive complaint according to the informant.

If No, →Negative cognitive complaint according to the informant.

**Supplementary material 2:** Domain-specific activities and the formula used to calculate functional impairment in activities of daily living.

| **Division of the 33 ADLs from the Technology–Activities of Daily Living into three functional domains** | |
| --- | --- |
| **1. Self-Care**  Eating  Dressing  Bathing  Elimination  Taking pills or medicine  Interest in personal appearance | BADL  BADL  BADL  BADL  IADL  BADL |
| **2. Household Care**  Preparing meals cooking  Setting the table  Housekeeping  Home maintenance  Home repairs  Laundry | IADL  IADL  IADL  IADL  IADL  IADL |
| **3. Employment and Recreation**  Employment  Recreation  Organization  Travel | AADL  AADL  AADL  AADL |
| **4. Shopping and Money**  Food shopping  Handling cash  Managing finances | IADL  IADL  IADL |
| **5. Travel**  Public transportation  Driving  Mobility around the neighborhood  Travel outside familiar environment | IADL  IADL  IADL  IADL |
| **6. Communication**  Using a telephone  Talking  Understanding  Reading  Writing | IADL  IADL  IADL  IADL  IADL |
| **7. Technology**  Computer use  Cell-phone use  ATM use  Internet access  Email access | AADL  IADL  IADL  AADL  AADL |

Functional impairment, expressed as a percentage, was calculated for each area, domain-specific subscore, and the overall ADL score using the following formula:

⅀ items scores (excluding ND / DK responses)

Functional = _____________________________________________________ X 100

decline (%)

3 x numbers of items answered (excluding ND /DK responses)

**Supplementary Table 1**. Two-way general linear models examining effects of diagnosis, sex, and diagnosis-by-sex interaction on total and domain-specific ADL performance.

| **Outcome** | **Diagnosis**  **F(df)** | ***p*-value** | **η²p** | **Sex**  **F(df)** | ***p*-value** | **η²p** | **Diagnosis×Sex**  **F(df)** | ***p*-value** | **η²p** |
| --- | --- | --- | --- | --- | --- | --- | --- | --- | --- |
| BADL | 36.71  (2,132) | <0.001 | 0.357 | 0.13  (1,132) | 0.722 | 0.001 | 0.39  (2,132) | 0.678 | 0.006 |
| IADL | 204.60  (2,132) | <0.001 | 0.756 | 0.72  (1,132) | 0.396 | 0.005 | 0.39  (2,132) | 0.675 | 0.006 |
| AADL | 29.83  (2,132) | <0.001 | 0.311 | 2,64  (1,132) | 0.106 | 0.020 | 1.19  (2,132) | 0.306 | 0.018 |
| Total ADL | 173.21  (2,132) | <0.001 | 0.724 | 2.08  (1,132) | 0.152 | 0.016 | 1.29  (2,132) | 0.279 | 0.019 |

Note: Two-way general linear models were performed including diagnosis (SCC, MCI, ADD), sex, and the diagnosis×sex interaction term. For each outcome, F values, degrees of freedom (df), p-values, and partial eta squared (η²p) are reported. Abbreviations: BADL, Basic Activities of Daily Living; IADL, Instrumental Activities of Daily Living; AADL, Advanced Activities of Daily Living; ADL, Activities of Daily Living; η²p, partial eta squared. Significance level: p < 0.05.

**Supplementary Table 2.** Areas of activities of daily living assessed by the T-ALDQ.

|  | **SCC (1)** | **MCI (2)** | **ADD (3)** | **𝝌^2^*/p* (global)** | ***p* (post-hoc)** |
| --- | --- | --- | --- | --- | --- |
| **Number of cases (138)** | 69 | 45 | 24 |  |  |
| Self-Care | 0.00 (0.00-0.00) | 0.00 (0.00-0.00) | 22.22 (12.50-27.78) | **<0.001^b^** | P1 =1.000 (1-2)  **P2 <0.001 (1-3)**  **P3 <0.001 (2-3)** |
| Household Care | 0.00 (0.00-11.11) | 0.00 (0.00-11.11) | 66.67 (46.67-78.33) | **<0.001^b^** | P1 =1.000 (1-2)  **P2 <0.001 (1-3)**  **P3 <0.001 (2-3)** |
| Employment and Recreation | 25.00 (0.00-33.33) | 33.33 (12.50-41.67) | 56.94 (33.33-66.67) | **<0.001^b^** | **P1 =0.038 (1-2)**  **P2 <0.001 (1-3)**  **P3 =0.003 (2-3)** |
| Shopping and Money | 0.00 (0.00-0.00) | 0.00 (0.00-0.00) | 55.55 (33.33-75.00) | **<0.001^b^** | P1 =0.378 (1-2)  **P2 <0.001 (1-3)**  **P3 <0.001 (2-3)** |
| Travel | 0.00 (0.00-9.72) | 8.33 (0.00-25.00) | 44.44 (25.00-66.67) | **<0.001^b^** | P1 =0.149 (1-2)  **P2 <0.001 (1-3)**  **P3 <0.001 (2-3)** |
| Communication | 0.00 (0.00-6.67) | 0.00 (0.00-12.22) | 36.67 (26.67-46.67) | **<0.001^b^** | P1 =0.756 (1-2)  **P2 <0.001 (1-3)**  **P3 <0.001 (2-3)** |
| Technology | 0.00 (0.00-16.67) | 22.22 (9.02-33.33) | 50.0 (33.33-70.00) | **<0.001^b^** | **P1 =0.002 (1-2)**  **P2 <0.001 (1-3)**  **P3 =0.001 (2-3)** |

Note: Data are presented as median (Q1–Q3) and mean ± standard deviation (minimum–maximum), except for sex, which is expressed as a percentage. a: Chi-square test; b: Kruskal–Wallis test (post hoc: Mann–Whitney U test); c: Welch one-way ANOVA (post hoc: Games–Howell); d: One-way ANOVA (post hoc: Tukey). Abbreviations: SCC: Subjective Cognitive Complaints; MCI: Mild Cognitive Impairment; ADD: Alzheimer’s Disease Dementia. P1: SCC vs. MCI; P2: SCC vs. ADD; P3: MCI vs. ADD. Significance level: *p* < 0.05.

**Supplementary Table 3.** Distribution of informant type across clinical group.

|  | **SCC (1)** | **MCI (2)** | **ADD (3)** | **𝝌^2^*/p* (global)** | ***p* (post-hoc)** |
| --- | --- | --- | --- | --- | --- |
| **Number of cases (n =138)** | 69 | 45 | 24 |  |  |
| **Informant type** |  |  |  |  |  |
| Spouse/partner | 22 (31.9%) | 15 (33.3%) | 11 (45.8%) | 0.465^a^ | -- |
| Child | 32 (46.4%) | 16 (35.6%) | 11 (45.8%) | 0.514 ^a^ | -- |
| Sibling | 3 (4.3%) | 4 (8.9%) | 0 (0.0%) | 0.282 ^a^ | -- |
| Friend | 6 (8.7%) | 3 (6.7%) | 0 (0.0%) | 0.475 ^a^ | **--** |
| Grandchild | 5 (7.2%) | 5 (11.1%) | 1 (4.2%) | 0.697 ^a^ | **--** |
| Niece/Nephew | 0 (0.0%) | 2 (4.4%) | 0 (0.0%) | 0.133 ^a^ | **--** |
| Daughter in law/son in law | 1 (1.4%) | 0 (0.0%) | 0 (0.0%) | 1.000 ^a^ | **--** |
| Nurse | 0 (0.0%) | 0 (0.0%) | 1 (4.2%) | 0.176 ^a^ | **--** |

Note: Data is expressed as a percentage. a: Chi-square test. Abbreviations: SCC, Subjective Cognitive Complaints; MCI, Mild Cognitive Impairment; ADD, Alzheimer’s Disease Dementia.

**Supplementary Figure 1:** Brain atrophy in the MCI group relative to the SCC group.


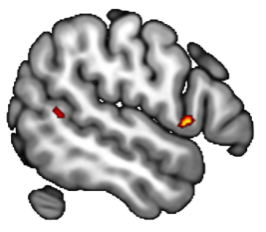


t

X = -52.5

Y = 10.5

Z = 3

R

L


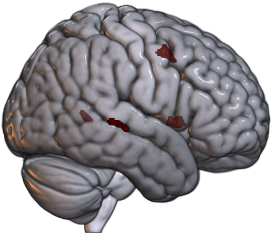


R


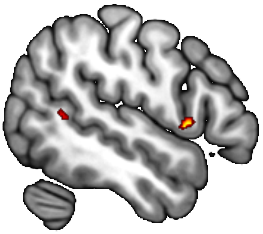

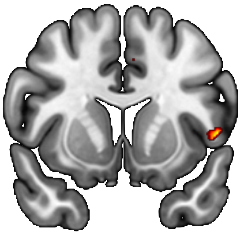


L


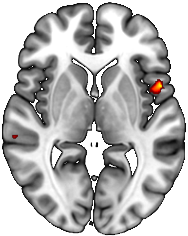

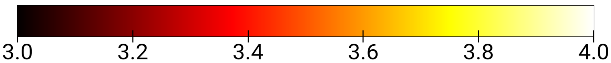


L

R

Coordinates are reported in MNI space (x, y, z). L: left; R: right. Color bars indicate *t*-values. All clusters for the MCI group are shown at p < 0.001 (uncorrected).

**Supplementary Table 4.** Gray matter atrophy in the MCI group relative to the SCC group.

| Cluster  N° Voxels | Peak *t* | Coordinates | | | Regions |
| --- | --- | --- | --- | --- | --- |
|  |  | x | y | z |  |
| 124 | 3,91 | -52,5 | 10,5 | 3 | Inferior frontal gyrus, opercular part L |
| 73 | 3,71 | -7,5 | 1,5 | 49,5 | Supplementary motor area L |
| 73 | 3,60 | 66 | -21 | -3 | Middle temporal gyrus R |
|  | 3,44 | 61,5 | -30 | 1,5 | Superior temporal gyrus R |
| 50 | 3,50 | -49,5 | -52,5 | 6 | Middle temporal gyrus L |

Atrophy differences were estimated at p < 0.001 (uncorrected). Coordinates are reported in MNI space (x, y, z). L: left; R: right.

**Supplementary Figure 2.** Brain atrophy in the ADD group relative to the SCC group.

t

X = -27

Y = -31.5

Z = -7.5

L

R

L


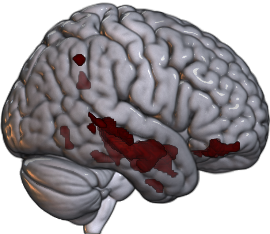

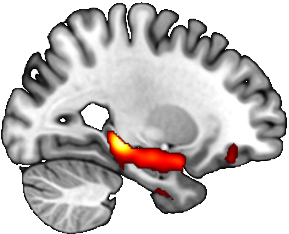

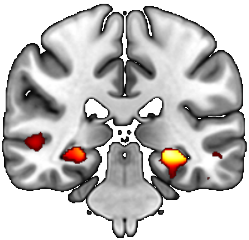


L

R

R


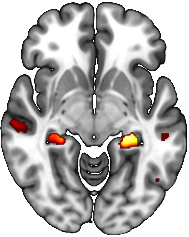

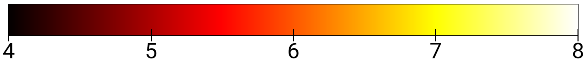


Coordinates are reported in MNI space (x, y, z). L: left; R: right. Color bars indicate t-values. All clusters for the ADD group are shown at p < 0.05 (FWE-corrected for multiple comparisons).

**Supplementary Table 5.** Gray matter atrophy in the ADD group relative to the SCC group.

| Cluster  N° Voxels | Peak *t* | Coordinates | | | Regions |
| --- | --- | --- | --- | --- | --- |
|  |  | x | y | z |  |
| 2573 | 7,75 | -27 | -31,5 | -7,5 | Hippocampus L |
|  | 6,00 | -21 | -6 | -15 | Amygdala L |
|  | 6,00 | -24 | 4,5 | -18 | Amygdala L |
| 1608 | 6,51 | 25,5 | -33 | -4,5 | Hippocampus R |
|  | 6,16 | 34,5 | -22,5 | -13,5 | Hippocampus R |
|  | 5,69 | 27 | -10,5 | -16,5 | Hippocampus R |
| 140 | 5,44 | -49,5 | -39 | -19,5 | Inferior temporal gyrus L |
|  | 4,65 | -49,5 | -31,5 | -22,5 | Inferior temporal gyrus L |
| 521 | 5,29 | 63 | -21 | -6 | Middle temporal gyrus R |
|  | 5,19 | 52,5 | -33 | 0 | Middle temporal gyrus R |
|  | 5,01 | 64,5 | -37,5 | 1,5 | Middle temporal gyrus R |
| 87 | 5,16 | -57 | -55,5 | 33 | Angular gyrus L |
| 62 | 5,15 | 52,5 | -51 | 43,5 | Inferior parietal gyrus R |
| 306 | 5,02 | 27 | 39 | -12 | Anterior orbital gyrus R |
| 163 | 5,01 | -27 | 36 | -16,5 | Anterior orbital gyrus L |
|  | 4,69 | -19,5 | 27 | -18 | Posterior orbital gyrus L |
| 96 | 4,98 | -30 | -3 | -40,5 | Fusiform gyrus L |
| 79 | 4,91 | -48 | -66 | -3 | Inferior occipital gyrus L |
|  | 4,76 | -48 | -63 | -12 | Inferior occipital gyrus L |
| 59 | 4,90 | -55,5 | -30 | -10,5 | Middle temporal gyrus L |

Atrophy differences were estimated at p < 0.001 and corrected for multiple comparisons using family-wise error (FWE) at p < 0.05. Coordinates are reported in MNI space (x, y, z). L: left; R: right.

**Supplementary Figure 3.** Brain atrophy in the ADD group relative to the MCI group.

t

X = 25.5

Y = -34.5

Z = -4.5


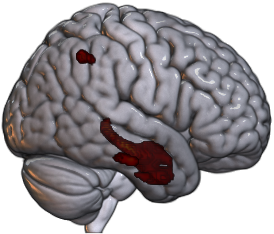

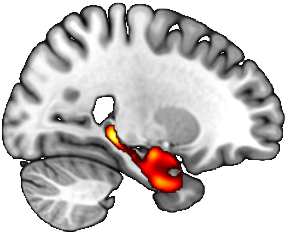

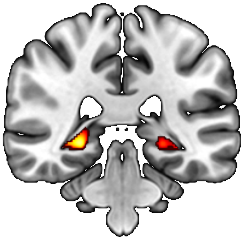

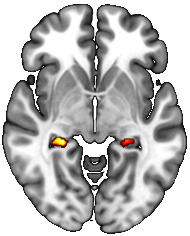

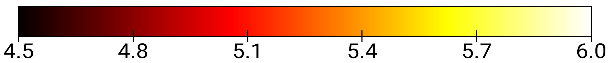


R

R

R

R

L

L

Coordinates are reported in MNI space (x, y, z). L: left; R: right. Color bars indicate t-values. All clusters for the ADD group are shown at p < 0.05 (FWE-corrected for multiple comparisons).

**Supplementary Table 6.** Gray matter atrophy in the ADD group relative to the MCI group.

| Cluster  N° Voxels | Peak *t* | Coordinates | | | Regions |
| --- | --- | --- | --- | --- | --- |
|  |  | x | y | z |  |
| 2356 | 5,87 | 25,5 | -34,5 | -4,5 | Hippocampus R |
|  | 5,77 | 37,5 | -21 | -15 | Parahippocampal gyrus R |
|  | 5,64 | 28,5 | -12 | -18 | Hippocampus R |
| 133 | 5,55 | 49,5 | -51 | 46,5 | Inferior parietal gyrus R |
| 333 | 5,45 | -34,5 | -31,5 | -9 | Hippocampus L |
|  | 5,43 | -25,5 | -31,5 | -6 | Parahippocampal gyrus L |
| 111 | 5,35 | 63 | -16,5 | -22,5 | Middle temporal gyrus R |
|  | 5,08 | 63 | -25,5 | -24 | Inferior temporal gyrus R |

Atrophy differences were estimated at p < 0.001 and corrected for multiple comparisons using family-wise error (FWE) at p < 0.05. Coordinates are reported in MNI space (x, y, z). L: left; R: right.

**Supplementary Figure 4.** Association between gray matter atrophy and total ADL performance in the MCI-SCC group.


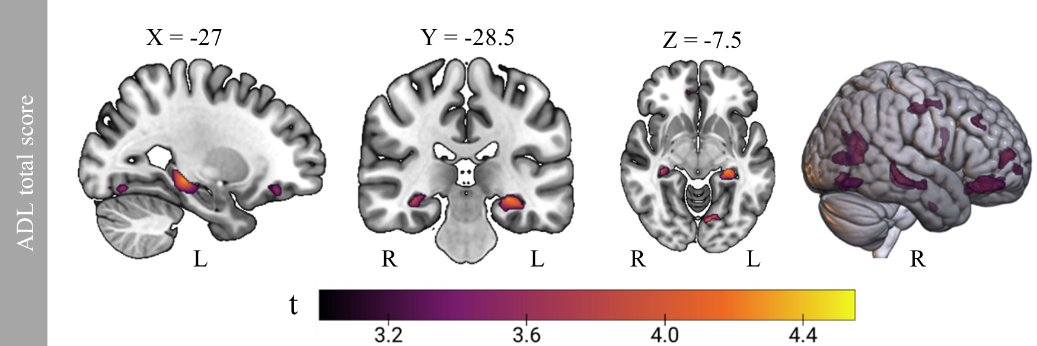


Coordinates are reported in MNI space (x, y, z). L: left; R: right. Color bars indicate *t*-values. All clusters for the MCI group are shown at p < 0.001 (uncorrected).

**Supplementary Table 7.** Brain regions associated with total ADL performance in the MCI-SCC group.

| Cluster  N° Voxels | Peak *t* | Coordinates | | | Regions |
| --- | --- | --- | --- | --- | --- |
|  |  | x | y | z |  |
| 443 | 4,53 | -15 | -78 | -10,5 | Lingual gyrus L |
|  | 3,78 | -31,5 | -76,5 | -12 | Fusiform gyrus L |
|  | 3,28 | -37,5 | -66 | -12 | Fusiform gyrus L |
| 1296 | 4,41 | -12 | -61,5 | 15 | Calcarine fissure and surrounding cortex L |
|  | 4,20 | -9 | -61,5 | 7,5 | Posterior cingulate L |
|  | 3,87 | -3 | -76,5 | 7,5 | Calcarine fissure and surrounding cortex L |
| 639 | 4,31 | -27 | -28,5 | -7,5 | Hippocampus L |
|  | 3,97 | -25,5 | -34,5 | -1,5 | Hippocampus L |
|  | 3,38 | -19,5 | -34,5 | 4,5 | Pulvinar medial L |
| 331 | 4,11 | -9 | -16,5 | 49,5 | Supplementary motor area L |
|  | 3,59 | -7,5 | 0 | 49,5 | Supplementary motor area L |
| 579 | 4,08 | 27 | 37,5 | -15 | Anterior orbital gyrus R |
|  | 3,62 | 25,5 | 48 | -18 | Anterior orbital gyrus R |
| 418 | 3,98 | 30 | -31,5 | -3 | Hippocampus R |
|  | 3,97 | 36 | -30 | -9 | Hippocampus R |
|  | 3,50 | 36 | -19,5 | -13,5 | Hippocampus R |
| 92 | 3,88 | 9 | 30 | 34,5 | Middle cingulate & paracingulate gyri R |
| 232 | 3,83 | 10,5 | 57 | 9 | Superior frontal gyrus, medial R |
|  | 3,75 | 7,5 | 51 | 3 | Superior frontal gyrus, medial R |
|  | 3,46 | 13,5 | 49,5 | 9 | Anterior cingulate cortex, pregenual R |
| 184 | 3,78 | -52,5 | 3 | 18 | Precentral gyrus L |
|  | 3,21 | -45 | 4,5 | 7,5 | Rolandic operculum L |
| 198 | 3,69 | -22,5 | 27 | -15 | Posterior orbital gyrus L |
|  | 3,61 | -28,5 | 36 | -16,5 | Anterior orbital gyrus L |
| 116 | 3,63 | -9 | 43,5 | -13,5 | Superior frontal gyrus, medial orbital L |
| 61 | 3,63 | 9 | 37,5 | -12 | Superior frontal gyrus, medial orbital R |
| 156 | 3,59 | 31,5 | -63 | -13,5 | Fusiform gyrus R |
|  | 3,57 | 31,5 | -72 | -13,5 | Fusiform gyrus R |
| 71 | 3,55 | -49,5 | -4,5 | -31,5 | Inferior temporal gyrus L |
| 109 | 3,47 | -15 | 61,5 | -1,5 | Superior frontal gyrus, medial L |

Regressions were estimated at p < 0.001 (uncorrected). Coordinates are reported in MNI space (x, y, z). L: left; R: right.

**Supplementary Figure 5.** Association between gray matter atrophy and total ADL performance in the ADD-SCC group.


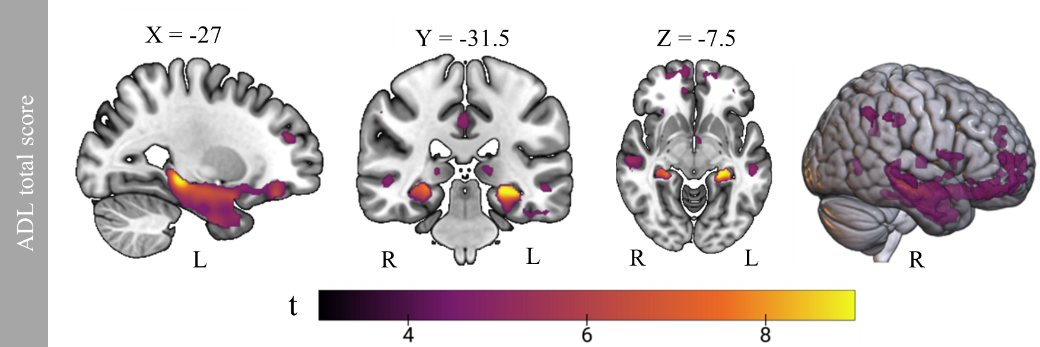


Coordinates are reported in MNI space (x, y, z). L: left; R: right. Color bars indicate *t*-values. All clusters for the ADD group are shown at p < 0.05 (FWE-corrected for multiple comparisons).

**Supplementary Table 8.** Brain regions associated with total ADL performance in the ADD-SCC group.

| Cluster  N° Voxels | Peak *t* | Coordinates | | | Regions |
| --- | --- | --- | --- | --- | --- |
|  |  | x | y | z |  |
| 5791 | 9,21 | -27 | -31,5 | -7,5 | Hippocampus L |
|  | 7,05 | -24 | 6 | -19,5 | Temporal pole: superior temporal gyrus L |
|  | 6,84 | -22,5 | -7,5 | -16,5 | Amygdala L |
| 2664 | 7,97 | 25,5 | -33 | -4,5 | Hippocampus R |
|  | 6,99 | 34,5 | -22,5 | -13,5 | Parahippocampal gyrus R |
|  | 6,53 | 27 | -10,5 | -16,5 | Hippocampus R |
| 1326 | 5,98 | 21 | 45 | -16,5 | Anterior orbital gyrus R |
|  | 5,84 | 27 | 39 | -12 | Anterior orbital gyrus R |
|  | 5,83 | 22,5 | 55,5 | 4,5 | Superior frontal gyrus, dorsolateral R |
| 173 | 5,82 | 52,5 | -51 | 43,5 | Inferior parietal gyrus R |
| 347 | 5,80 | -18 | 64,5 | 4,5 | Superior frontal gyrus, dorsolateral L |
|  | 5,22 | -12 | 58,5 | -6 | Superior frontal gyrus, dorsolateral L |
|  | 4,91 | -19,5 | 58,5 | -3 | Superior frontal gyrus, dorsolateral L |
| 71 | 5,55 | -48 | -3 | -37,5 | Inferior temporal gyrus L |
|  | 4,70 | -51 | -7,5 | -30 | Fusiform gyrus L |
| 98 | 5,52 | 1,5 | 9 | -3 | Olfactory cortex R |
|  | 4,74 | -3 | -3 | -7,5 | Hypothalamus |
| 135 | 5,48 | 9 | 40,5 | -10,5 | Superior frontal gyrus, medial orbital R |
| 100 | 5,47 | -57 | -55,5 | 33 | Angular gyrus L |
| 392 | 5,43 | 60 | -21 | -6 | Superior temporal gyrus R |
|  | 4,91 | 51 | -31,5 | -1,5 | Middle temporal gyrus R |
| 178 | 5,36 | -25,5 | 45 | 21 | Superior frontal gyrus, dorsolateral L |
|  | 5,24 | -18 | 46,5 | 30 | Superior frontal gyrus, dorsolateral L |
| 78 | 5,23 | 58,5 | -39 | 42 | Supramarginal gyrus R |
| 109 | 5,21 | -3 | -12 | 9 | Mediodorsal medial magnocellular L |
|  | 4,80 | -4,5 | -15 | 1,5 | Mediodorsal medial magnocellular L |
| 154 | 5,19 | -52,5 | -30 | -9 | Middle temporal gyrus L |
|  | 4,82 | -57 | -19,5 | -16,5 | Middle temporal gyrus L |
| 155 | 5,05 | 1,5 | -31,5 | 39 | Middle cingulate & paracingulate gyri R |
| 115 | 5,04 | -48 | -66 | 0 | Middle temporal gyrus L |
| 85 | 4,99 | 12 | 66 | 10,5 | Superior frontal gyrus, medial R |
| 236 | 4,97 | -37,5 | 6 | 9 | Inferior frontal gyrus, opercular part L |
|  | 4,69 | -30 | 15 | 10,5 | Insula L |
| 68 | 4,90 | -55,5 | -19,5 | -25,5 | Inferior temporal gyrus L |
|  | 4,76 | -64,5 | -12 | -24 | Middle temporal gyrus L |
| 138 | 4,87 | 37,5 | 3 | 13,5 | Insula R |
|  | 4,73 | 34,5 | 18 | 10,5 | Insula R |

Regressions were estimated at p < 0.001 and corrected for multiple comparisons using family-wise error (FWE) at p < 0.05. Coordinates are reported in MNI space (x, y, z). L: left; R: right.

**Supplementary Table 9.** Brain regions associated with AADL performance in the MCI-SCC group.

| Cluster  N° Voxels | Peak *t* | Coordinates | | | Regions |
| --- | --- | --- | --- | --- | --- |
|  |  | x | y | z |  |
| 168 | 4,38 | -15 | -76,5 | -9 | Lingual gyrus L |
| 184 | 4,34 | 9 | 37,5 | -12 | Superior frontal gyrus, medial orbital R |
|  | 3,30 | 12 | 46,5 | -6 | Superior frontal gyrus, medial orbital R |
| 652 | 4,29 | 22,5 | 25,5 | -15 | Medial orbital gyrus R |
|  | 3,87 | 31,5 | 39 | -9 | Middle frontal gyrus R |
| 144 | 3,99 | -43,5 | 1,5 | 7,5 | Insula L |
| 129 | 3,90 | 54 | -39 | -4,5 | Middle temporal gyrus R |
| 177 | 3,76 | 27 | -75 | -16,5 | Fusiform gyrus R |
|  | 3,42 | 31,5 | -63 | -13,5 | Fusiform gyrus R |
| 196 | 3,75 | -24 | 28,5 | -13,5 | Posterior orbital gyrus L |
| 163 | 3,70 | -13,5 | 61,5 | 0 | Superior frontal gyrus, medial L |
| 246 | 3,66 | -6 | -15 | 48 | Supplementary motor area L |
|  | 3,64 | -10,5 | -6 | 45 | Middle cingulate & paracingulate gyri L |
|  | 3,41 | -7,5 | 1,5 | 46,5 | Supplementary motor area L |
| 108 | 3,61 | 28,5 | -30 | -4,5 | Hippocampus R |
| 62 | 3,57 | -4,5 | -79,5 | 6 | Calcarine fissure and surrounding cortex L |
| 74 | 3,46 | 25,5 | 54 | 9 | Superior frontal gyrus, dorsolateral R |
| 70 | 3,42 | -51 | -37,5 | 0 | Middle temporal gyrus L |
|  | 3,28 | -57 | -48 | 6 | Middle temporal gyrus L |
| 52 | 3,33 | -24 | -31,5 | -6 | Hippocampus L |
| 54 | 3,32 | -9 | -61,5 | 7,5 | Posterior cingulate gyrus L |

Regressions were estimated at p < 0.001 (uncorrected). Coordinates are reported in MNI space (x, y, z). L: left; R: right.

**Supplementary Table 10.** Brain regions associated with AADL performance in the ADD-SCC group.

| Cluster  N° Voxels | Peak *t* | Coordinates | | | Regions |
| --- | --- | --- | --- | --- | --- |
|  |  | x | y | z |  |
| 617 | 6,81 | -22,5 | -33 | -7,5 | Parahippocampal gyrus L |
|  | 6,65 | -36 | -31,5 | -10,5 | Hippocampus L |
| 441 | 6,54 | 25,5 | -31,5 | -6 | Hippocampus R |
| 806 | 5,79 | -28,5 | 37,5 | -18 | Anterior orbital gyrus L |
|  | 5,44 | -24 | 9 | -21 | Posterior orbital gyrus L |
|  | 4,94 | -19,5 | 30 | -16,5 | Posterior orbital gyrus L |
| 74 | 5,44 | 9 | 40,5 | -10,5 | Superior frontal gyrus, medial orbital R |
| 252 | 5,44 | -18 | 64,5 | 3 | Superior frontal gyrus, dorsolateral L |
|  | 5,28 | -12 | 58,5 | -4,5 | Superior frontal gyrus, medial orbital L |
| 91 | 5,15 | -27 | 46,5 | 21 | Middle frontal gyrus L |
| 272 | 5,15 | 22,5 | 37,5 | -16,5 | Anterior orbital gyrus R |
| 105 | 4,97 | -39 | 1,5 | 12 | Insula L |
| 132 | 4,77 | 25,5 | -9 | -16,5 | Hippocampus R |

Regressions were estimated at p < 0.001 and corrected for multiple comparisons using family-wise error (FWE) at p < 0.05. Coordinates are reported in MNI space (x, y, z). L: left; R: right.

**Supplementary Table 11.** Brain regions associated with IADL performance in the MCI-SCC group.

| Cluster  N° Voxels | Peak *t* | Coordinates | | | Regions |
| --- | --- | --- | --- | --- | --- |
|  |  | x | y | z |  |
| 899 | 4,83 | -30 | -30 | -10,5 | Hippocampus L |
|  | 3,61 | -19,5 | -34,5 | 4,5 | Pulvinar medial thalamus L |
| 1090 | 4,48 | -13,5 | -61,5 | 15 | Calcarine fissure and surrounding cortex L |
|  | 3,70 | -3 | -76,5 | 6 | Calcarine fissure and surrounding cortex L |
|  | 3,69 | -12 | -75 | 25,5 | Cuneus L |
| 499 | 4,27 | -34,5 | -75 | -16,5 | Fusiform gyrus L |
|  | 3,60 | -31,5 | -58,5 | -13,5 | Fusiform gyrus L |
| 887 | 4,16 | 30 | -12 | -16,5 | Hippocampus R |
|  | 4,15 | 30 | -31,5 | -3 | Hippocampus R |
|  | 4,13 | 36 | -28,5 | -10,5 | Hippocampus R |
| 136 | 4,11 | -15 | -78 | -10,5 | Lingual gyrus L |
| 356 | 4,01 | -54 | 3 | 18 | Precentral gyrus L |
|  | 3,69 | -57 | -1,5 | 7,5 | Rolandic operculum L |
|  | 3,66 | -55,5 | 6 | 27 | Precentral gyrus L |
| 532 | 3,95 | 27 | 37,5 | -15 | Anterior orbital gyrus R |
|  | 3,90 | 25,5 | 49,5 | -18 | Anterior orbital gyrus R |
| 197 | 3,90 | 12 | 54 | 4,5 | Superior frontal gyrus, medial R |
| 99 | 3,77 | -9 | -16,5 | 49,5 | Supplementary motor area L |
| 68 | 3,75 | -48 | -57 | 7,5 | Middle temporal gyrus L |
| 85 | 3,74 | 25,5 | 7,5 | -18 | Olfactory cortex R |
| 89 | 3,69 | 10,5 | 31,5 | 34,5 | Middle cingulate & paracingulate gyri R |
| 103 | 3,55 | 31,5 | -72 | -12 | Fusiform gyrus R |
|  | 3,44 | 31,5 | -63 | -12 | Fusiform gyrus R |
| 135 | 3,55 | -49,5 | -4,5 | -31,5 | Inferior temporal gyrus L |
| 77 | 3,52 | -28,5 | 36 | -16,5 | Anterior orbital gyrus L |
|  | 3,32 | -22,5 | 30 | -13,5 | Posterior orbital gyrus L |
| 51 | 3,51 | 42 | -81 | -1,5 | Inferior occipital gyrus R |
| 60 | 3,34 | -9 | 43,5 | -12 | Superior frontal gyrus, medial orbital L |

Regressions were estimated at p < 0.001 (uncorrected). Coordinates are reported in MNI space (x, y, z). L: left; R: right.

**Supplementary Table 12.** Brain regions associated with IADL performance in the ADD-SCC group.

| Cluster  N° Voxels | Peak *t* | Coordinates | | | Regions |
| --- | --- | --- | --- | --- | --- |
|  |  | x | y | z |  |
| 5607 | 9,38 | -31,5 | -30 | -10,5 | Hippocampus L |
|  | 7,24 | -24 | 6 | -19,5 | Temporal pole: superior temporal gyrus L |
|  | 7,16 | -22,5 | -9 | -16,5 | Hippocampus L |
| 2835 | 7,89 | 25,5 | -33 | -3 | Hippocampus R |
|  | 7,26 | 33 | -22,5 | -13,5 | Hippocampus R |
|  | 6,06 | 21 | -3 | -15 | Amygdala R |
| 1328 | 5,97 | 22,5 | 55,5 | 4,5 | Superior frontal gyrus, dorsolateral R |
|  | 5,93 | 21 | 45 | -16,5 | Anterior orbital gyrus R |
|  | 5,69 | 27 | 39 | -12 | Anterior orbital gyrus R |
| 467 | 5,90 | -48 | -4,5 | -37,5 | Inferior temporal gyrus L |
|  | 5,35 | -55,5 | -19,5 | -25,5 | Inferior temporal gyrus L |
|  | 5,15 | -51 | -7,5 | -30 | Fusiform gyrus L |
| 292 | 5,71 | -48 | -40,5 | -19,5 | Inferior temporal gyrus L |
|  | 5,15 | -52,5 | -31,5 | -22,5 | Inferior temporal gyrus L |
|  | 4,60 | -51 | -52,5 | -16,5 | Inferior temporal gyrus L |
| 53 | 5,69 | 4,5 | -12 | 9 | Mediodorsal medial magnocellular R |
| 132 | 5,62 | 51 | -51 | 43,5 | Inferior parietal gyrus R |
| 156 | 5,51 | 1,5 | 7,5 | -3 | Anterior cingulate & paracingulate gyri R |
|  | 5,00 | -3 | -1,5 | -7,5 | Hypothalamus |
| 249 | 5,50 | -18 | 64,5 | 4,5 | Superior frontal gyrus, dorsolateral L |
|  | 5,04 | -12 | 57 | -9 | Superior frontal gyrus, medial orbital L |
|  | 4,85 | -19,5 | 58,5 | -3 | Superior frontal gyrus, dorsolateral L |
| 212 | 5,38 | 61,5 | -19,5 | -6 | Superior temporal gyrus R |
| 155 | 5,31 | -46,5 | -64,5 | 0 | Middle temporal gyrus L |
| 121 | 5,28 | -3 | -12 | 9 | Mediodorsal medial magnocellular L |
|  | 4,87 | -4,5 | -15 | 1,5 | Mediodorsal medial magnocellular L |
| 141 | 5,20 | -18 | 46,5 | 30 | Superior frontal gyrus, dorsolateral L |
|  | 5,15 | -25,5 | 45 | 21 | Superior frontal gyrus, dorsolateral L |
| 77 | 5,18 | 9 | 42 | -10,5 | Superior frontal gyrus, medial orbital R |
| 66 | 5,01 | 58,5 | -39 | 42 | Supramarginal gyrus R |
| 88 | 4,90 | 1,5 | -30 | 39 | Middle cingulate & paracingulate gyri R |
| 94 | 4,82 | -39 | 9 | 3 | Insula L |
|  | 4,66 | -30 | 15 | 9 | Insula L |
| 61 | 4,81 | -42 | -13,5 | -7,5 | Superior temporal gyrus L |
|  | 4,72 | -36 | -21 | 3 | Heschl's gyrus L |

Regressions were estimated at p < 0.001 and corrected for multiple comparisons using family-wise error (FWE) at p < 0.05. Coordinates are reported in MNI space (x, y, z). L: left; R: right.

**Supplementary Table 13.** Brain regions associated with BADL performance in the ADD-SCC group.

| Cluster  N° Voxels | Peak *t* | Coordinates | | | Regions |
| --- | --- | --- | --- | --- | --- |
|  |  | x | y | z |  |
| 144 | 4,88 | -33 | -28,5 | -12 | Hippocampus L |
| 72 | 4,86 | 40,5 | 6 | 12 | Insula R |

Regressions were estimated at p < 0.001 and corrected for multiple comparisons using family-wise error (FWE) at p < 0.05. Coordinates are reported in MNI space (x, y, z). L: left; R: right.

**Supplementary Figure 6.** Association between gray matter atrophy and technology-related functional performance in the MCI-SCC group.

t

Technology performance

X = 9

Y = -60

Z = -1.5


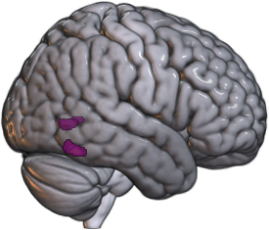


R


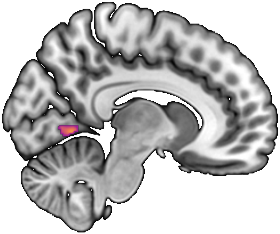


R


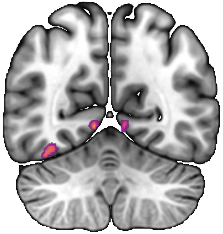


R


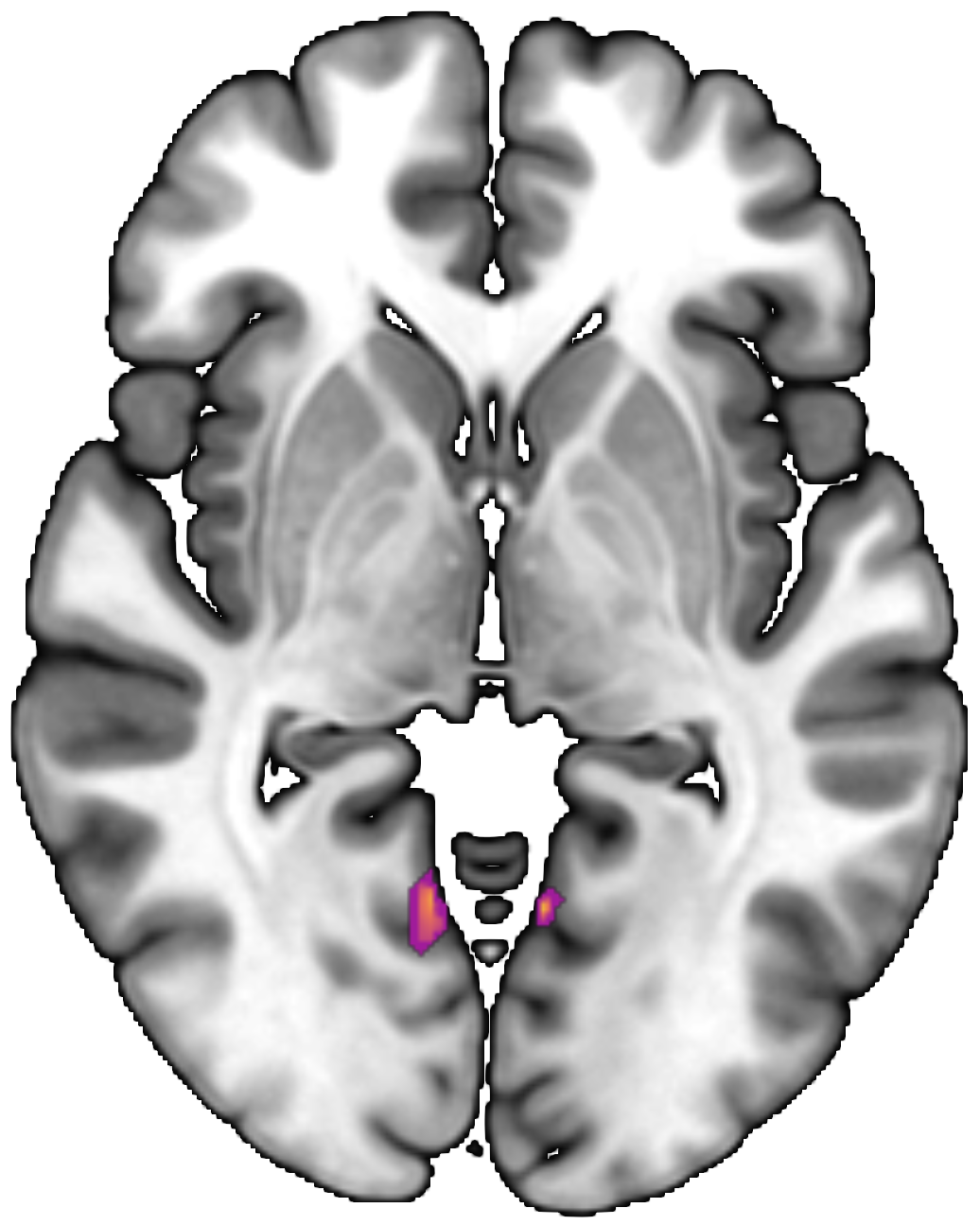


L

L

R


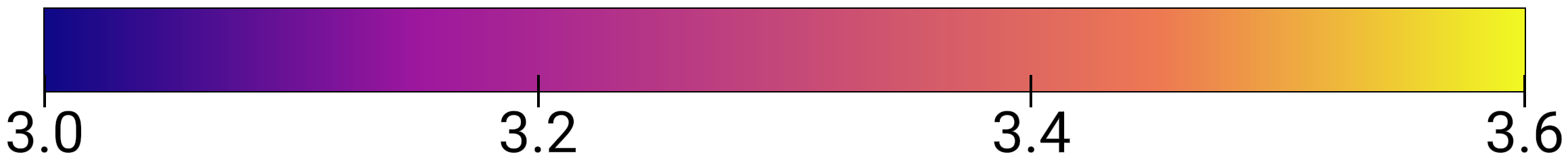


Coordinates are reported in MNI space (x, y, z). L: left; R: right. Color bars indicate *t*-values. All clusters for the MCI group are shown at p < 0.001 (uncorrected).

**Supplementary Table 14.** Brain regions associated with technology-related functional performance in the MCI-SCC group.

| Cluster  N° Voxels | Peak *t* | Coordinates | | | Regions |
| --- | --- | --- | --- | --- | --- |
|  |  | x | y | Z |  |
| 56 | 3,57 | -7,5 | -61,5 | -1,5 | Lingual gyrus L |
|  | 3,35 | -10,5 | -52,5 | 1,5 | Lingual gyrus L |
| 90 | 3,51 | 9 | -60 | -1,5 | Lingual gyrus R |
| 169 | 3,46 | 39 | -58,5 | -18 | Fusiform gyrus R |

Regressions were estimated at p < 0.001 (uncorrected). Coordinates are reported in MNI space (x, y, z). L: left; R: right.

**Supplementary Table 15.** Brain regions exclusively associated with AADL performance in the MCI-SCC group.

| Cluster  N° Voxels | Peak *t* | Coordinates | | | Regions |
| --- | --- | --- | --- | --- | --- |
|  |  | x | y | z |  |
| 168 | 4,38 | -15 | -76,5 | -9 | Lingual gyrus L |
| 117 | 4,34 | 9 | 37,5 | -12 | Superior frontal gyrus, medial orbital R |
| 458 | 4,29 | 22,5 | 25,5 | -15 | Medial orbital gyrus R |
|  | 3,89 | 24 | 37,5 | -12 | Anterior orbital gyrus R |
|  | 3,87 | 31,5 | 39 | -9 | Middle frontal gyrus R |
| 103 | 3,99 | -43,5 | 1,5 | 7,5 | Insula L |
| 129 | 3,90 | 54 | -39 | -4,5 | Middle temporal gyrus R |
| 177 | 3,76 | 27 | -75 | -16,5 | Fusiform gyrus R |
|  | 3,42 | 31,5 | -63 | -13,5 | Fusiform gyrus R |
| 89 | 3,75 | -24 | 28,5 | -13,5 | Posterior orbital gyrus L |
|  | 3,43 | -19,5 | 22,5 | -18 | Medial orbital gyrus |
| 246 | 3,66 | -6 | -15 | 48 | Supplementary motor area L |
|  | 3,64 | -10,5 | -6 | 45 | Middle cingulate & paracingulate gyri L |
|  | 3,41 | -7,5 | 1,5 | 46,5 | Supplementary motor area L |
| 62 | 3,57 | -4,5 | -79,5 | 6 | Calcarine fissure and surrounding cortex L |
| 74 | 3,46 | 25,5 | 54 | 9 | Superior frontal gyrus, dorsolateral R |
| 70 | 3,42 | -51 | -37,5 | 0 | Middle temporal gyrus L |
|  | 3,28 | -57 | -48 | 6 | Middle temporal gyrus L |
| 54 | 3,32 | -9 | -61,5 | 7,5 | Calcarine fissure and surrounding cortex L |

Regressions were estimated at p < 0.001 (uncorrected). Coordinates are reported in MNI space (x, y, z). L: left; R: right.

**Supplementary Table 16.** Brain regions exclusively associated with AADL performance in the ADD-SCC group.

| Cluster  N° Voxels | Peak *t* | Coordinates | | | Regions |
| --- | --- | --- | --- | --- | --- |
|  |  | x | y | Z |  |
| 527 | 6,46 | -21 | -34,5 | -7,5 | Parahippocampal gyrus L |
|  | 6,39 | -36 | -33 | -10,5 | Hippocampus L |
| 346 | 6,30 | 25,5 | -33 | -7,5 | Parahippocampal gyrus R |
| 338 | 5,79 | -28,5 | 37,5 | -18 | Anterior orbital gyrus L |
|  | 4,88 | -16,5 | 45 | -18 | Medial orbital gyrus L |
| 51 | 5,44 | -18 | 64,5 | 3 | Superior frontal gyrus, dorsolateral L |
| 335 | 5,44 | -24 | 9 | -21 | Posterior orbital gyrus L |
|  | 4,68 | -22,5 | -4,5 | -16,5 | Amygdala L |
| 80 | 5,28 | -12 | 58,5 | -4,5 | Superior frontal gyrus, medial orbital L |
| 91 | 5,15 | -27 | 46,5 | 21 | Middle frontal gyrus L |
| 71 | 5,07 | 21 | 37,5 | -18 | Anterior orbital gyrus R |
| 59 | 4,93 | -39 | 0 | 12 | Insula L |
| 132 | 4,77 | 25,5 | -9 | -16,5 | Hippocampus R |

Regressions were estimated at p < 0.001 and corrected for multiple comparisons using family-wise error (FWE) at p < 0.05. Coordinates are reported in MNI space (x, y, z). L: left; R: right.

**Supplementary Table 17.** Overlapping brain regions associated with AADL performance in both the MCI-SCC and ADD-SCC groups.

| Cluster  N° Voxels | Peak *t* | Coordinates | | | Regions |
| --- | --- | --- | --- | --- | --- |
|  |  | x | y | Z |  |
| 63 | 6,81 | -22,5 | -33 | -7,5 | Parahippocampal gyrus L |
| 95 | 6,54 | 25,5 | -31,5 | -6 | Hippocampus R |
| 133 | 5,57 | -28,5 | 36 | -16,5 | Anterior orbital gyrus L |
|  | 5,20 | -22,5 | 15 | -18 | Posterior orbital gyrus L |
|  | 4,94 | -19,5 | 30 | -16,5 | Posterior orbital gyrus L |
| 65 | 5,44 | 9 | 40,5 | -10,5 | Superior frontal gyrus, medial orbital R |
| 118 | 5,43 | -18 | 63 | 3 | Superior frontal gyrus, dorsolateral L |
|  | 5,22 | -12 | 60 | -3 | Superior frontal gyrus, medial orbital L |
| 201 | 5,15 | 22,5 | 37,5 | -16,5 | Anterior orbital gyrus R |

Regressions were estimated at p < 0.001 and corrected for multiple comparisons using family-wise error (FWE) at p < 0.05. Coordinates are reported in MNI space (x, y, z). L: left; R: right.

**Supplementary Table 18.** Brain regions exclusively associated with IADL performance in the MCI-SCC group.

| Cluster  N° Voxels | Peak *t* | Coordinates | | | Regions |
| --- | --- | --- | --- | --- | --- |
|  |  | x | y | Z |  |
| 1090 | 4,48 | -13,5 | -61,5 | 15 | Calcarine fissure and surrounding cortex L |
|  | 3,70 | -3 | -76,5 | 6 | Calcarine fissure and surrounding cortex L |
|  | 3,69 | -12 | -75 | 25,5 | Cuneus L |
| 499 | 4,27 | -34,5 | -75 | -16,5 | Fusiform gyrus L |
|  | 3,60 | -31,5 | -58,5 | -13,5 | Fusiform gyrus L |
| 52 | 4,15 | 30 | -31,5 | -3 | Hippocampus R |
| 136 | 4,11 | -15 | -78 | -10,5 | Lingual gyrus L |
| 356 | 4,01 | -54 | 3 | 18 | Precentral gyrus L |
|  | 3,69 | -57 | -1,5 | 7,5 | Rolandic operculum L |
|  | 3,66 | -55,5 | 6 | 27 | Precentral gyrus L |
| 179 | 3,90 | 25,5 | 49,5 | -18 | Anterior orbital gyrus R |
|  | 3,73 | 27 | 37,5 | -10,5 | Inferior frontal gyrus, orbitalis part R |
|  | 3,47 | 28,5 | 39 | -19,5 | Anterior orbital gyrus R |
| 187 | 3,90 | 12 | 54 | 4,5 | Superior frontal gyrus, medial R |
| 99 | 3,77 | -9 | -16,5 | 49,5 | Supplementary motor area L |
| 68 | 3,75 | -48 | -57 | 7,5 | Middle temporal gyrus L |
| 89 | 3,69 | 10,5 | 31,5 | 34,5 | Middle cingulate & paracingulate gyri R |
| 57 | 3,68 | 27 | 9 | -18 | Insula R |
| 103 | 3,55 | 31,5 | -72 | -12 | Fusiform gyrus R |
|  | 3,44 | 31,5 | -63 | -12 | Fusiform gyrus R |
| 87 | 3,55 | -49,5 | -4,5 | -31,5 | Inferior temporal gyrus L |
| 51 | 3,51 | 42 | -81 | -1,5 | Inferior occipital gyrus R |
| 60 | 3,34 | -9 | 43,5 | -12 | Superior frontal gyrus, medial orbital L |

Regressions were estimated at p < 0.001 (uncorrected). Coordinates are reported in MNI space (x, y, z). L: left; R: right.

**Supplementary Table 19.** Brain regions exclusively associated with IADL in the ADD-SCC group.

| Cluster  N° Voxels | Peak *t* | Coordinates | | | Regions |
| --- | --- | --- | --- | --- | --- |
|  |  | x | y | z |  |
| 4599 | 8,13 | -19,5 | -37,5 | -4,5 | Parahippocampal gyrus L |
|  | 7,24 | -27 | -12 | -16,5 | Hippocampus L |
|  | 7,24 | -24 | 6 | -19,5 | Temporal pole: superior temporal gyrus L |
| 1938 | 7,61 | 25,5 | -30 | -9 | Hippocampus R |
|  | 7,31 | 22,5 | -36 | -1,5 | Parahippocampal gyrus R |
|  | 6,91 | 34,5 | -19,5 | -18 | Hippocampus R |
| 700 | 5,97 | 22,5 | 55,5 | 4,5 | Superior frontal gyrus, dorsolateral R |
|  | 5,23 | 25,5 | 52,5 | -12 | Middle frontal gyrus R |
|  | 5,17 | 12 | 58,5 | -10,5 | Superior frontal gyrus, medial orbital |
| 162 | 5,91 | 19,5 | 45 | -18 | Anterior orbital gyrus R |
|  | 5,22 | 21 | 34,5 | -18 | Medial orbital gyrus R |
| 406 | 5,90 | -48 | -4,5 | -37,5 | Inferior temporal gyrus L |
|  | 5,35 | -55,5 | -19,5 | -25,5 | Inferior temporal gyrus L |
|  | 5,10 | -54 | -28,5 | -9 | Middle temporal gyrus L |
| 292 | 5,71 | -48 | -40,5 | -19,5 | Inferior temporal gyrus L |
|  | 5,15 | -52,5 | -31,5 | -22,5 | Inferior temporal gyrus L |
|  | 4,60 | -51 | -52,5 | -16,5 | Inferior temporal gyrus L |
| 53 | 5,69 | 4,5 | -12 | 9 | Mediodorsal medial magnocellular R |
| 132 | 5,62 | 51 | -51 | 43,5 | Inferior parietal gyrus R |
| 156 | 5,51 | 1,5 | 7,5 | -3 | Anterior cingulate & paracingulate gyri R |
|  | 5,00 | -3 | -1,5 | -7,5 | Hypothalamus |
| 248 | 5,50 | -18 | 64,5 | 4,5 | Superior frontal gyrus, dorsolateral L |
|  | 5,04 | -12 | 57 | -9 | Superior frontal gyrus, medial orbital L |
|  | 4,85 | -19,5 | 58,5 | -3 | Superior frontal gyrus, dorsolateral L |
| 212 | 5,38 | 61,5 | -19,5 | -6 | Superior temporal gyrus R |
| 155 | 5,31 | -46,5 | -64,5 | 0 | Middle temporal gyrus L |
| 121 | 5,28 | -3 | -12 | 9 | Mediodorsal medial magnocellular L |
|  | 4,87 | -4,5 | -15 | 1,5 | Mediodorsal medial magnocellular L |
| 140 | 5,20 | -18 | 46,5 | 30 | Superior frontal gyrus, dorsolateral L |
|  | 5,15 | -25,5 | 45 | 21 | Superior frontal gyrus, dorsolateral L |
| 77 | 5,18 | 9 | 42 | -10,5 | Superior frontal gyrus, medial orbital R |
| 66 | 5,01 | 58,5 | -39 | 42 | Supramarginal gyrus R |
| 88 | 4,90 | 1,5 | -30 | 39 | Middle cingulate & paracingulate gyri R |
| 94 | 4,82 | -39 | 9 | 3 | Insula L |
|  | 4,66 | -30 | 15 | 9 | Insula L |
| 61 | 4,81 | -42 | -13,5 | -7,5 | Superior temporal gyrus L |
|  | 4,72 | -36 | -21 | 3 | Heschl’s gyrus L |

Regressions were estimated at p < 0.001 and corrected for multiple comparisons using family-wise error (FWE) at p < 0.05. Coordinates are reported in MNI space (x, y, z). L: left; R: right.

**Supplementary Table 20.** Overlapping brain regions associated with IADL performance in both the MCI-SCC and ADD-SCC groups.

| Cluster  N° Voxels | Peak *t* | Coordinates | | | Regions |
| --- | --- | --- | --- | --- | --- |
|  |  | x | y | z |  |
| 899 | 9,38 | -31,5 | -30 | -10,5 | Hippocampus L |
| 863 | 7,89 | 25,5 | -33 | -3 | Hippocampus R |
|  | 7,26 | 33 | -22,5 | -13,5 | Hippocampus R |
|  | 5,65 | 22,5 | -13,5 | -25,5 | Parahippocampal gyrus R |
| 106 | 6,08 | -25,5 | 36 | -16,5 | Anterior orbital gyrus L |
| 380 | 5,93 | 21 | 45 | -16,5 | Anterior orbital gyrus R |
|  | 5,69 | 27 | 39 | -12 | Anterior orbital gyrus R |
| 61 | 5,88 | -48 | -4,5 | -36 | Inferior temporal gyrus L |
|  | 4,93 | -52,5 | -9 | -28,5 | Inferior temporal gyrus L |

Regressions were estimated at p < 0.001 and corrected for multiple comparisons using family-wise error (FWE) at p < 0.05. Coordinates are reported in MNI space (x, y, z). L: left; R: right.
